# Disulfiram with Cu^2+^ alleviates dextran sulfate sodium-induced ulcerative colitis in mice

**DOI:** 10.7150/thno.81571

**Published:** 2023-05-08

**Authors:** Wei Zhou, Hua Zhang, Lihua Huang, Chuankai Sun, Yuhan Yue, Xiaolei Cao, Hongling Jia, Chunyue Wang, Yunfei Gao

**Affiliations:** 1 The Fifth Affiliated Hospital (Heyuan Shenhe People's Hospital), Jinan University, Heyuan, Guangdong, China.; 2 The Biomedical Translational Research Institute, Faculty of Medical Science, Jinan University, Guangzhou, Guangdong, China.; 3 Department of Metabolic and Bariatric Surgery, The First Affiliated Hospital of Jinan University, Guangzhou, Guangdong, China.; 4 Laboratory of Pathogenic Biology and Immunology, College of Basic Medical, Inner Mongolia Medical University, Hohhot, China.; 5 Department of Medical Biochemistry and Molecular Biology, School of Medicine, Jinan University, Guangzhou, Guangdong, China.; 6 Fuwai Hospital, National Center for Cardiovascular Diseases, State Key Laboratory of Cardiovascular Disease, Chinese Academy of Medical Sciences and Peking Union Medical College, Beijing, China.

**Keywords:** ulcerative colitis, disulfiram, macrophage, CD4^+^ T cells, microbiota

## Abstract

**Background:** Disulfiram (DSF), a Food and Drug Administration (FDA)-approved drug for chronic alcohol addiction, has anti-inflammatory effects that help prevent various cancers, and Cu^2+^ can enhance the effects of DSF. Inflammatory bowel diseases (IBD) are characterized by chronic or recurrent relapsing gastrointestinal inflammation. Many drugs targeting the immune responses of IBD have been developed, but their application has many problems, including side effects and high costs. Therefore, there is an urgent need for new drugs. In this study, we investigated the preventive effects of DSF+Cu^2+^ on dextran sulfate sodium (DSS)-induced ulcerative colitis (UC) in mice.

**Methods:** The anti-inflammatory effects were investigated using the DSS-induced colitis mouse model and lipopolysaccharide (LPS)-induced macrophages. DSS-induced TCRβ^-/-^ mice were used to demonstrate the effect of DSF in conjunction with Cu^2+^ on CD4^+^ T cell-secreted interleukin 17 (IL-17). In addition, the effect of DSF+Cu^2+^ on intestinal flora was studied by 16S rRNA microflora sequencing.

**Results:** DSF and Cu^2+^ could significantly reverse the symptom of DSS-induced UC in mice, such as weight loss, disease activity index score, colon length shortening, and reversal of colon pathological changes. DSF and Cu^2+^ could inhibit colonic macrophage activation by blocking the nuclear factor kappa B (NF-κB) pathway, reducing nucleotide-binding oligomerization domain, leucine-rich repeat and pyrin domain-containing 3 (NLRP3)-inflammasome-derived interleukin 1 beta (IL-1β) secretion and caspase-1 (CASP1) activation, and decreasing IL-17 secretion by CD4^+^ T cells. Moreover, the treatment of DSF and Cu^2+^ could protect the intestinal barrier by reversing the expression of tight junction proteins, zonula occluden-1 (ZO-1), occludin, and mucoprotein-2 (MUC2). Additionally, DSF+Cu^2+^ could reduce the abundance of harmful bacteria and increase beneficial bacteria in the intestinal tract of mice, effectively improving intestinal microecology.

**Conclusion:** Our study evaluated the effect of DSF+Cu^2+^ on the immune system and gut microbiota in colonic inflammation and highlighted its potential to treat UC in the clinic.

## Introduction

The incidence rate of inflammatory bowel disease (IBD), an intractable autoimmune disease, is increasing worldwide 1. There are two main phenotypes of IBD: Crohn's disease (CD) and ulcerative colitis (UC). The symptoms of UC patients include abdominal pain, diarrhea, repeated attacks, and prolonged treatment time, seriously affecting the quality of life 2. Although the exact pathogenesis of UC is unclear, it is generally believed that the causes include genetic background, environmental factors, gut microbiota, and immunological dysfunction 3, 4. Many conventional drugs for UC, including corticosteroids, amino-salicylates, antibiotics 5, and antitumor necrosis factor α (TNF-α) therapies, have been developed and are universally used in clinical settings 6. However, their applications have many problems, including side effects, high recurrence rates, single effects and high cost. Thus, the development of new therapeutics is of utmost importance.

Disulfiram (tetraethylthiuram disulfide, DSF), has been used for over six decades as a treatment for alcohol dependence 7. Its pharmacokinetics, safety, and tolerability are well established at the US Food and Drug Administration (FDA) - recommended doses 8. DSF chelates bivalent metals and forms complexes with copper (Cu), which enhances its anti-tumor activity 9, 10. DSF is anti-inflammatory and has cancer prevention effects 11. Previous studies have shown that DSF can prevent and treat diet-induced obesity and block pyroptosis of cells and septic death induced by lipopolysaccharide (LPS) in mice. Furthermore, Cu^2+^ supplementation may enhance DSF efficacy after LPS induction 12-14. However, it is unclear whether DSF+Cu^2+^ prevents the development or progression of intestinal inflammation by altering the interaction between the immune system and gut microbes.

The intestinal mucosa of UC patients produces various of proinflammatory factors, such as tumor necrosis factor (TNF), interleukin-6 (IL-6), and interleukin-1β (IL-1β) 15. The complex interaction among intestinal microbes, intestinal epithelial cells and resident immune cells actively contributes to the stability of the gastrointestinal environment 16. Abnormal expression of MUC2 by goblet cells and tight junction proteins (ZO-1 and occludin) in intestinal tissues can induce immune dysfunction 17. The epithelial barrier is the first line of defense against bacterial invasion. Damage to the epithelial barrier leads to increased colon permeability, resulting in changes in the innate and acquired host immune response 16. UC involves the infiltration of inflammatory cells, including macrophages, neutrophils, T cells, and other lymphocytes. Among all the inflammatory cells, macrophages have been the most studied in UC pathogenesis 18. In the absence of inflammation, dendritic cells are mainly involved in antigen presentation, while macrophages secrete less pro-inflammatory cytokines. During inflammation, cytokines responsible for macrophage activation are secreted. Macrophages can be classically activated (M1) or alternately activated (M2). M1 macrophages have pro-inflammatory properties and can be activated by LPS and secrete many cytokines (TNF-α, IL-1β, IL-6, IL-18, IL-23) in response to stimulation. M1 macrophages are involved in the immune response through Th1 and Th17 cells, and M2 macrophages show anti-inflammatory function 19. NF-κB promotes transcription of the genes encoding proinflammatory cytokines, such as TNF-α, IL1β, and IL-6, considered to be related to the development and pathogenesis of UC 20. The inflammasome is a multi-protein complex composed of nucleotide-binding oligomerization domain, leucine-rich repeat and pyrin domain-containing 3 (NLRP3), ASC (apoptosis-associated speck-like protein containing a caspase recruitment domain (CARD)), and pro-caspase-1. Activation of the inflammasome leads to proteolytic activation of caspase-1, then cleaves the cytokine precursors pro-IL-1β and pro-IL-18 into mature IL-1β and IL-18 21. Many studies also demonstrated that NLRP3 inflammasome-mediated IL-1β release and CASP1 activation were involved in experimental colitis 22. NLRP3 can be activated by NF-κB as a first step in pathway activation, both of which regulate the balance between mucosal homeostasis and inflammation in colitis. This suggests that inhibiting macrophage NF-κB signaling and NLRP3 inflammasome activation might improve dextran sulfate sodium (DSS)-induced colitis. Inflammation promotes lymphocyte activation due to the increased activity of antigen-presenting cells such as macrophages in UC. CD4^+^ T cells polarize into Th17 cells in the presence of IL-6, TGF-β, IL-1β, and IL-23. IL-1β, IL-6, and TGF-β secreted by macrophages mediate the activation of RORγt, which is a key transcription factor for naive CD4^+^ T cells to convert to Th17 cells. UC patients showed an increase in interleukin 17 (IL-17)-related cytokines associated with an increased number of Th17 cells 23. Th17 cells secrete IL-17A and IL-17F. IL-17A stimulates the secretion of chemokines by monocytes, epithelial cells, and endothelial cells, which attract lymphocytes to inflamed tissues, thereby causing an inflammatory response in the gut 24.

The intestinal flora is composed of various of microbes, which are believed to be crucial in gut movement, intestinal barrier function, and the occurrence of UC 25. The unsteady interaction between intestinal flora and host mucosal immunity induces abnormal immune responses against symbiotic nonpathogenic bacteria 23. It has been reported that intestinal microbiota disorder could affect the balance of regulatory T (Treg)/T helper type 17 (Th17), thereby triggering intestinal inflammation 26, 27. In this study, we evaluated the effect of DSF and Cu^2+^ on the immune system and gut microbiota on colonic inflammation and investigated the mechanism for their actions by using peritoneal macrophage, J774A.1, THP-1, and BMDM-based assay system *in vitro* and DSS-induced colitis mice model *in vivo*.

## Materials and Methods

### Mice

WT and TCRβ^-/-^ mice on C57BL/6J background were purchased from The Jackson Laboratory and inbred at our university facility. The mice were fed with food and water ad libitum in standard animal cages kept in a room at 22 ± 1 °C with a 12 h light/dark cycle under specific pathogen-free conditions at Jinan University (Guangzhou, China). The experimental colitis mice model was established by administering 2.5% (w/v) DSS, MW, 36,000-50,000 kDa (MP Biochemicals, Aurora, OH, USA) dissolved in standard drinking water (days 0-7). Mice were randomly divided into three groups: the normal group received drinking water, the DSS group received 2.5% DSS, and the DSF and Cu^2+^ group received Cu^2+^ (0.15 mg/kg) intragastrically 6 h before intragastrical administration of DSF (50 mg/kg) along with DSS administration. Cu^2+^ solution was prepared by dissolving in PBS and DSF was formulated in 10% DMSO and 90% corn oil. Mice were administered intragastrically with 200 μL of Cu^2+^ (0.15 mg/kg) and DSF (50 mg/kg) solution at the specified concentration once daily for 7 days. An equal volume of PBS and corn oil was given to mice in normal and DSS groups. For adoptive transfer of naive CD4^+^ T cells, TCRβ^-/-^ recipient mice were transferred with naive CD4^+^ T cells (2 × 106) pretreated with DSF and Cu^2+^ (suspended in 200 μL sterile PBS) or not via ophthalmic vein injection. Control TCRβ^-/-^ mice were injected with 200 μL PBS. Two days later, the reconstituted mice were induced with DSS for colitis. At the end of the experiment, their colons were excised and photographed, and the length of the colon was measured from the ileocecal junction to the anal verge. All animal procedures were executed in accordance with the guidelines for the Institutional Animal Care and Use Committee of Jinan University.

### Disease activity index (DAI) assessment

DAI was calculated daily for each mouse, including body weight loss, gross bleeding, and stool consistency. DAI scores were calculated as the mean value of the three indexes. The scoring principles of DAI were as follows: (a) body weight loss, none = 0; 1-5% = 1; 5-10% = 2; 10-20% = 3; over 20% = 4; (b) gross bleeding, negative = 0; positive = 2; gross rectal bleeding = 4; (c) stool consistency, normal = 0; loose stools = 2; diarrhea = 4 27.

### Histopathological examination

Mice were executed on day 7, colons were collected and photographed, and colon length was measured. Excised colonic tissues were fixed in 4% paraformaldehyde, embedded in paraffin, and stained with hematoxylin and eosin (H&E) for histological examination. Histological evaluation of H&E-stained colonic sections was graded as follows: (a) severity of inflammation, none = 0; mild = 1; moderate = 2; severe = 3; (b) crypt damage, none = 0; basal 1/3 damaged = 1; basal 2/3 damaged = 2; only surface epithelium intact = 3; entire crypt and epithelium lost = 4; (c) sites of the lesion, none = 0; mucosal layer = 1; submucosal layer = 2; muscle layer = 3; transmural = 4; The histological scores were shown as the sum of the three evaluations with a maximal score of 11.22 27.

### Preliminary evaluation of biosafety

Mice were divided into four groups: (i) Normal (ii) Cu^2+^ (0.15 mg/kg) (iii) DSF (50 mg/kg) (iv) Cu^2+^ (0.15 mg/kg) + DSF (50 mg/kg). Mice were executed on day 7, and the heart, liver, spleen, and kidney were collected, fixed in 4% paraformaldehyde, embedded in paraffin, and stained with H&E for histological examination.

### Isolation of lymphocytes from colonic lamina propria

Colonic tissues were cut into 1 cm segments and collected in 50 mL tubes. Tissue fragments were incubated in dissociation buffer (RPMI 1640 supplemented 2% FBS, 1 mM EDTA, 1 mM DTT, and 10 mM HEPES) for 30 min at 37 °C using a shaker to remove epithelial cells. The residual parts were incubated in the digestion buffer (RPMI 1640 supplemented 5% FBS, 10 μg/mL DNase I, and 1 mg/mL collagenase IV) for 45 min at 37 °C with shaking. The suspension was filtered through a 70-micron mesh, and centrifuged at 1200 r.p.m. for 20 min. Enrichment of lymphocytes from lamina propria was performed using 35% and 75% Percoll gradients.

### Flow cytometry

Cells were incubated with specific antibodies for 15 min at 4 °C in the dark for cell surface staining. For intracellular staining, cells were stimulated with 50 ng/ml phorbol 12-myristate 13-acetate (PMA, Sigma) and 1 μg/ml ionomycin (MCE) in the presence of GolgiStop (BD Biosciences) for 4 h. Subsequently, cells were fixed and permeabilized with BD Cytofix/Cytoperm Plus (BD Biosciences), and incubated with specific antibodies for another 30 min at 4 °C in the dark. All samples were acquired with FACSVerse flow cytometer (BD Biosciences) and analyzed with FlowJo software (TreeStar). The following antibodies were used: PE-CY7-conjugated anti-mouse F4/80 (BioLegend), PerCP-conjugated anti-mouse CD11b (BioLegend), APC-CY7-conjugated anti-mouse CD45RA (BioLegend), PerCP-conjugated anti-mouse CD3 (BioLegend), PE-conjugated anti-mouse CD4 (BioLegend), APC-conjugated anti-mouse CD4 (BioLegend), Bv421-conjugated anti-mouse IL-17A (BioLegend), PE-conjugated anti-mouse IL-17A (BioLegend).

### *In vitro* mouse T cell differentiation

Mouse naive splenic CD4^+^ T cells were prepared using the Naive CD4^+^ T Cell Isolation Kit (STEMCELL Technologies). Naive CD4^+^ T cells were activated by plate-bound anti-mouse CD3 (10 μg/mL) and anti-mouse CD28 (1 μg/mL) for 4 days with the following cytokines and antibodies: TGF-β1.2 (2 ng/mL, R&D Systems), IL-6 (40 ng/mL, PeproTech), IL-1β (10 ng/mL, PeproTech), IL-23 (20 ng/mL, PeproTech), anti-IFN-γ (10 μg/mL, BioLegend) and anti-IL-4 (10 μg/mL, BioLegend) for Th17. We cultured cells in 48-well plates with a total volume of 0.5 ml/well of culture medium with 2.5 × 10^5^ cells.

### Cell preparation and stimulation

J774A.1 macrophages were purchased from SUYAN (Guangzhou, Guangdong, China) and were cultured in DMEM (Gibco, USA) supplemented with 10% fetal bovine serum (FBS, Gibco, USA) and 100 U/ml penicillin/streptomycin (Gibco, USA) at 37 °C and 5% CO_2_. Human THP-1 monocytic cells (ELGBIO) were grown in RPMI 1640 (Gibco, USA) supplemented with 10% FBS and 100 U/ml penicillin/streptomycin at 37 °C and 5% CO_2_. THP-1 cells were differentiated 48 h with 50 ng/ml PMA. Bone marrow-derived macrophages (BMDMs) were obtained from the bone marrow cells of the femur and tibia of mice. The harvested cells were incubated in DMEM, supplemented with 10% FBS, 100 U/ml penicillin/streptomycin, and 20 ng/mL murine macrophage colony-stimulating factor (M-CSF) at 37 °C and 5% CO_2_ for 6 days to induce differentiation. Mice were intraperitoneally injected with 2 mL of 4% thioglycollate solution (SIGMA) and sacrificed 4 days after the injection. Their peritoneal cavities were washed with RPMI 1640 and centrifuged at 300 g for 10 min. The cell pellet was suspended in RPMI 1640 containing 10% FBS and 100 U/ml penicillin/streptomycin. Cells (1.2×10^6^ cells/well) were seeded in 12-well plates, incubated at 37 °C for two hours, and washed with RPMI 1640 with 10% FBS and 100 U/ml penicillin/streptomycin. The attached cells (1.0×10^6^ cells/well) were used as macrophages. The anti-inflammatory effects of test agents were measured following treating macrophages with LPS (1 μg/ml or 100 ng/mL) in the absence or presence of test agents for 90 min (NF-κB signaling molecules) or 24 h (inducible NO synthase (iNOS), cyclooxygenase-2 (COX-2), and cytokines). J774A.1 and THP-1 cells were primed with 1 μg/mL LPS for 5 h and BMDMs were primed with 100 ng/ml LPS for 3.5 h before stimulation with nigericin (10 μM 1 h) or adenosine triphosphate (ATP) (5 mM 45 min). The effect of bis (diethyldithiocarbamate)-copper (CuET) was evaluated by adding the indicated concentration of CuET to cell culture medium 4 h before stimulating with nigericin or ATP.

### RNA extraction, cDNA synthesis, and quantitative real-time PCR (qPCR)

Cells were harvested with 500 μL RZ (TIANGEN Biotech), and RNA was extracted with the RNA Simple Total RNA Kit (TIANGEN Biotech) according to the manufacturer's protocols. Colonic tissue RNA was extracted with the RNeasy Mini Kit (QIAGEN GmbH) according to the manufacturer's instructions. 200-500 ng of RNA from each sample was reverse-transcribed with the PrimeScript RT Reagent Kit (Takara). cDNA was diluted at 1:5 or 1:10 in RNase/DNAse-free water for further analysis by qPCR. QRT-PCR reactions were run in duplicate on the CFX Connect System (Bio-Rad). 2× SYBR Green qPCR Master Mix (Bimake) was used according to the manufacturer's instructions. Each qPCR reaction consisted of a 20 µL final volume. The running conditions were: 5 minutes at 95 °C, followed by 45 cycles of 5 seconds at 95 °C and 30 seconds at 60 °C, and then 15 seconds at 95 °C, 1 minute at 60 °C, and 15 seconds at 95 °C. After each run, a melting curve was obtained for each PCR product to control for primer dimers and gene-specific peaks. The internal reference was GAPDH. The specificity of the PCR products was confirmed by melting curve analysis. The list of primer sequences is provided in Table S1.

### Enzyme-linked immunosorbent assay (ELISA)

The supernatant of colonic tissue homogenate was collected to detect the protein levels of TNF-α, IL-1β, and IL-6. After the total protein concentration of the supernatant was determined, specific ELISA kits (BioLegend) were used to detect TNF-α, IL-1β, and IL-6. The final values were calculated by normalizing the protein mass (pg) of TNF-α, IL-1β, and IL-6 to the total protein mass (mg).

### Immunoblotting analysis

Protein was harvested from colonic tissues or cells with RIPA Lysis Buffer (Beyotime) supplemented with phenylmethyl sulfonyl fluoride (PMSF) protease inhibitor and phosphatase inhibitor. Nuclear proteins from cultured cells were extracted using Nuclear and Cytoplasmic Extraction Reagents (Boster). Cell supernatants from 6 wells were pooled and centrifuged at 4 °C for 5 min at 2,000 g. Pelleted debris was discarded, and the supernatant was concentrated by TCA precipitation. Total proteins (20-40 µg) were separated on 10% or 12% sodium dodecyl sulfate-polyacrylamide gels and transferred onto 0.22 µm PVDF membrane with the PowerPac wet-blot system (Bio-Rad). Membranes were incubated in blocking solution (5% BSA or 5% skim milk) for 1 hour, then incubated with the indicated primary antibodies overnight at 4 ℃. Primary antibodies included anti-occludin (Abcam), anti-ZO-1 (Abcam), anti-COX2 (CST), anti-iNOS (Abcam), anti-p-IκB (Abcam), anti-IκB (Abcam), anti-p-p65 (CST), anti-p65 (CST), anti-NLRP3 (Adipogen), anti-ASC (Santa), anti-caspase1 (Adipogen), anti-IL-1β (Adipogen), anti-lamin B1 (Abcam), and anti-β-actin (CST). After 6 washes in TBST for 1 h, the membranes were incubated with secondary antibodies conjugated to HRP at a dilution of 1:4,000 (CST, goat anti-rabbit, 7074 or goat anti-mouse, 7076) at room temperature for 2 h. Finally, membranes were washed 5 times in TBST, and enhanced chemiluminescence (Millipore) was used to record the chemiluminescence signals on Bio-Rad ChemiDoc MP Gel imaging system (Bio-Rad). Images were quantified by Image J analysis software.

### Immunohistochemistry

Colonic tissues were fixed in 4% (w/v) paraformaldehyde solution, embedded in paraffin, sectioned, dewaxed, and then treated with the antigen. Next, the samples were incubated with primary and secondary antibodies at 4 °C. Subsequently, the nuclei were stained with DAPI, and the sections were imaged using a Leica TCS SP8 confocal microscope.

### Immunofluorescence histochemistry

Briefly, the sections were deparaffinized, rehydrated and washed in 1% PBS-Tween20, treated with 2% hydrogen peroxide, blocked with 3% goat serum and incubated for 2 h at room temperature with anti-CD11b FITC (1: 100). The slides were then counter-stained with DAPI for 2 min, and finally washed in water for 20 min. Images were acquired by Leica TCS SP8 confocal microscope. F4/80 and IL-1β antibodies were used to detect F4/80^+^ IL-1β cells and F4/80 and p65 antibodies were used to detect F4/80^+^ p65 cells. Settings for image acquisition were identical for control and experimental tissues.

### Terminal deoxynucleotidyl transferase dUTP nick-end labeling analysis (TUNEL assay)

Colonic apoptosis was visualized on paraffin-embedded sections using a TUNEL assay (*In situ* Cell Death Detection kit, Roche, Germany) according to the manufacturer's instructions. Fluorescent images were captured on a Leica TCS SP8 confocal microscope.

### FACS staining for activated CASP1

Cells of colonic tissues were isolated from normal and drug-treated colitis mice at day 7 and stained with CD11b-APC and FLICA CASP1 (FITC) according to the operation protocol (FLICA CASP1 assay Kit, Immunochemistry Technologies Company). Activation of CASP1 in CD11b^+^ cells was analyzed by FACS.

### Fecal genomic DNA extraction and 16S-rRNA sequencing

The genomic DNA of the samples was extracted by cetyltrimethylammonium bromide or SDS, and then the purity and concentration of DNA were detected by agarose gel electrophoresis. An appropriate amount of DNA in a centrifuge tube was diluted to 1 ng/μL with sterile water. Amplicon libraries covering the V4 hypervariable regions of the bacterial 16S-rDNA gene were amplified using primers 515F: 5′-GTGCCAGCMGCCGCGGTAA-3′, and 806R: 5′-GGACTACHVGGGTWTCTAAT-3′. PCR was performed using a 25 μL mixture containing 5 μL of 5× buffer, 2 μL of 2.5 mM dNTPs, 1 μL of each primer (5 μM), 0.25 μL of Fast Pfu polymerase, 1 μL of template DNA, 14.75 μL of ddH_2_O. PCR was conducted as follows: initial denaturation for 3 min at 95 °C followed by 27 cycles of 30 s at 95 °C, 30 s for annealing at 55 °C, 45 s for elongation at 72 °C, and a final extension at 72 °C for 10 min. PCR products were detected by electrophoresis on 2% agarose gel. Qualified PCR products were purified by the magnetic beads and quantified by enzyme-labeled method. The target band was recovered using the gel recovery kit provided by Qiagen. The library was constructed using TruSeq Preparation DNA PCR-Free sample preparation kit, quantified using Qubit and Q-PCR, and sequenced using NovaSeq 6,000.

### Statistical analyses

Statistical analysis was conducted using GraphPad Prism 9.3.1. Where indicated, data were analyzed for statistical significance and reported as p-values. Data were analyzed by two-tailed Student's *t*-test when comparing means of two independent groups and two-way ANOVA when comparing more than two groups. For sample sizes of *n* > 3, the data distribution was first checked using the Kolmogorov-Smirnov test. If the data fitted a normal distribution, a two-tailed unpaired Student's *t*-test was used when variances were similar, whereas a two-tailed unpaired Student's *t*-test with Welch's correction was used when variances were different. If the data did not fit a normal distribution, a Mann-Whitney U-test was used. p < 0.05 was considered statistically significant (*, *p* < 0.05; **, *p* < 0.01; ***, *p* < 0.001; and ****, *p* < 0.0001). Data were presented as the mean ± SD.

## Results

### DSF+Cu^2+^ alleviated DSS-induced colitis in mice

**Figure 1:**
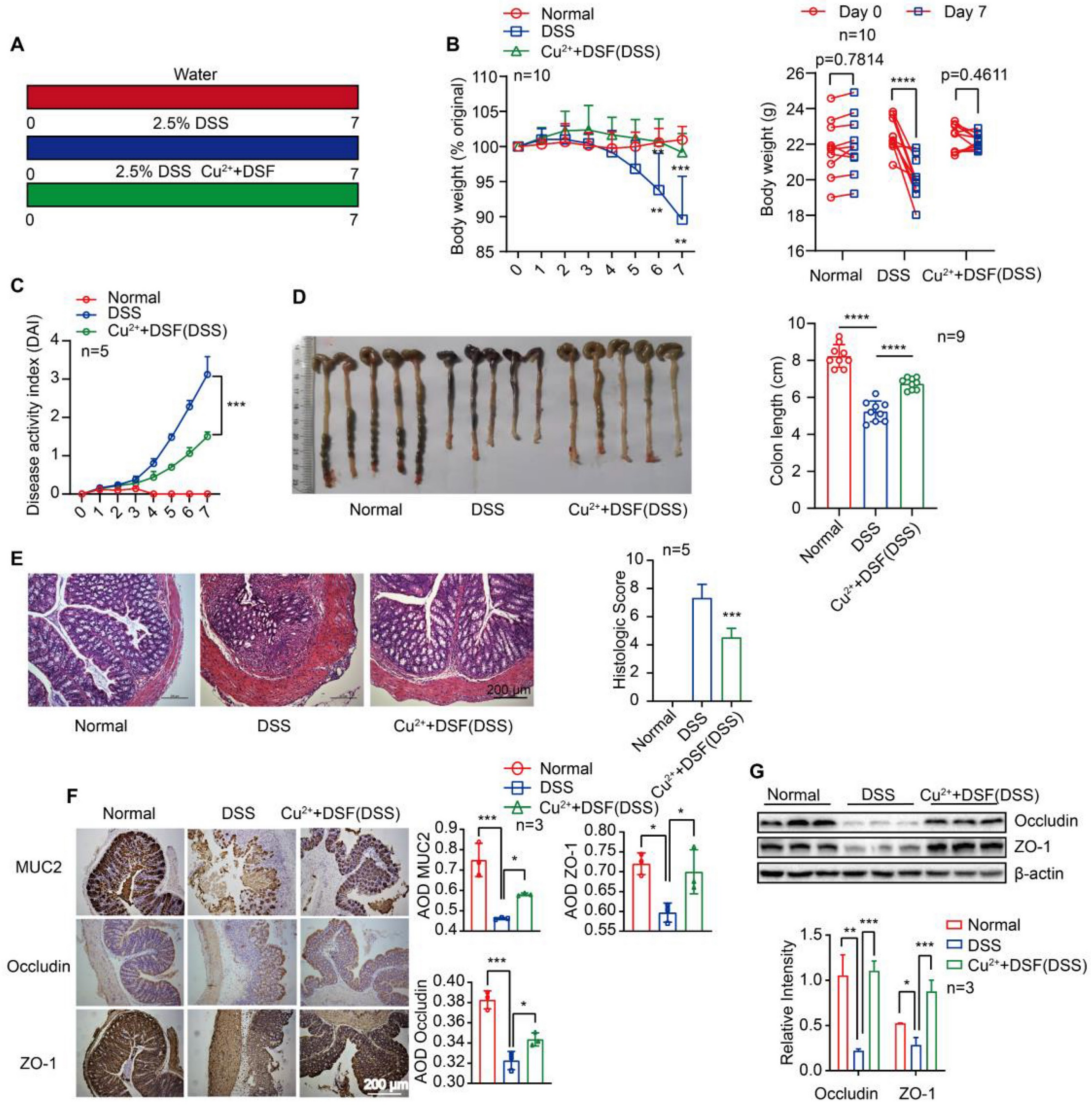
** DSF+Cu^2+^ alleviated DSS-induced colitis in mice.** (A) Mice were orally administered with 2.5% DSS and Cu^2+^ and DSF for 7 consecutive days. Mice were then sacrificed, and colons were collected. (B) Body weight change. (C) DAI score. (D) Colon length. (E) Histological changes were detected using H&E staining (scale bar, 200 μm). (F) Representative immunostaining images of colon sections stained for MUC2, ZO-1 and occludin (scale bar, 200 μm). (G) Representative Western blots of occludin and ZO-1 expression in the colon. Data were presented as the mean ± SD and represented 1 of at least 2 independent experiments with consistent results. One-way ANOVA with Tukey's multiple comparisons test (B-G) was used to calculate statistical significance (*p< 0.05, **p < 0.01, ***p < 0.001, and ****p < 0.001).

For our study, we administered doses of DSF (50 mg/kg) or copper gluconate (0.15 mg/kg) that were equivalent to 284 mg/day or 0.85 mg/day in humans, respectively, after adjusting for differences in body surface area using allometric scaling. The doses of disulfiram used in our study fell within the clinically approved range of 125-500 mg/day for treating alcohol dependence, while the doses of copper gluconate ranged from 0.5-1.5 mg/day when orally administered to adults 14, 28. The side effects and toxicities of DSF+Cu^2+^ were evaluated using histopathological examination, showing no noticeable pathological changes in the major organs of each group after drug administration every day for 7 days **(Figure S1)**. Histological analysis of mouse intestinal epithelial cells revealed no obvious pathological changes, no inflammatory cell infiltration, intact crypts, no mucosal damage, and no reduction in the number of crypts **(Figure S2A)**. To evaluate epithelial damage and integrity, we assessed the numbers of apoptotic cells and epithelial cell proliferation using TUNEL and Ki-67 staining, respectively. There was no significant change in the numbers of apoptotic cells in the colon epithelial cells of the treated mice, when compared among the four groups of mice **(Figure S2B-C)**. Furthermore, there was no change in the numbers of actively proliferating epithelial cells after treatment with the drug, as indicated by the Ki67-positive proliferating cells in the colon **(Figure S2D-E)**. These results suggest that DSF or Cu^2+^ alone or in combination did not cause any noticeable toxic effect on intestinal epithelial cells. We first investigated whether DSF+Cu^2+^ could be used to treat colitis in an animal model induced by DSS. In the control group, mice received water for continuous 7 days, the model group received 2.5% DSS, and the experimental group received 2.5% DSS with DSF and Cu^2+^ **(Figure 1A)**. In the model group, DSS-challenged mice manifested severe colitis, indicated by a dramatic increase in DAI scores evaluated by body weight loss, bloody stool, and stool consistency. Compared with the DSS group, DSF and Cu^2+^ treatment significantly attenuated body weight loss and reduced DAI score during disease progression **(Figure 1B-C)**. DSS typically causes colonic shortening observed in DSS-challenged mice. Oral administration of DSF and Cu^2+^ prevented the colonic shortening induced by DSS **(Figure 1D)**. Histological analysis showed serious epithelial disruption, inflammatory cell infiltration, distortion of crypts, mucous membrane damage, and decreased crypt numbers in colitis mice. These histopathological abnormities were largely restored in mice treated with DSF and Cu^2+^ **(Figure 1E)**.

The expression of MUC2 and tight junction proteins ZO-1 and occludin was significantly decreased in the colons of DSS-induced mice. However, the expression of these proteins increased significantly following treatment with DSF and Cu^2+^ **(Figure 1F-G)**. We also investigated whether Cu^2+^ alone has the protective effects or enhance the effectiveness of DSF in treating colitis. Our results showed that compared to Cu^2+^ or DSF alone, the combination of DSF+Cu^2+^ significantly reduced body weight loss, decreased DAI score, and alleviated DSS-induced colonic shortening **(Figure S3)**. Thus, the administration of DSF partially protected mice from DSS-induced colitis, while simultaneous administration of Cu^2+^ improved its therapeutic effects. These results showed that DSF+Cu^2+^ could alleviate the development and progression of colitis.

### DSF+Cu^2+^ inhibited DSS-induced proinflammatory cytokine expression, NF-κB signaling, and CASP1 activation in UC mice

**Figure 2:**
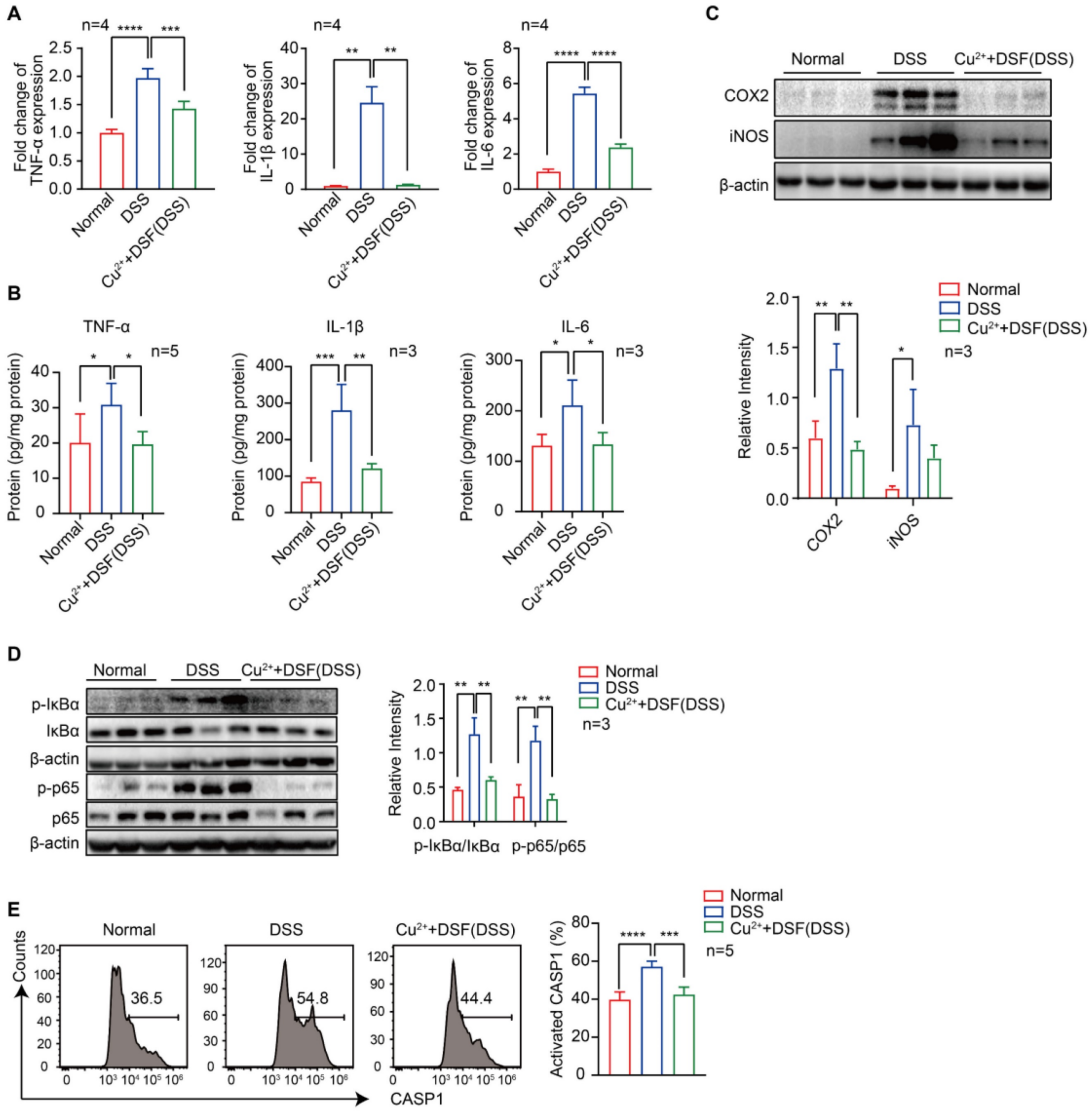
** DSF+Cu^2+^ inhibited DSS-induced proinflammatory cytokine expression, NF-κB signaling, and CASP1 activation in UC mice.** Mice were orally administered with 2.5% DSS and Cu^2+^ and DSF for 7 consecutive days. Mice were then sacrificed, and colons were collected. (A) mRNA levels of TNF-α, IL-1β, and IL-6 in colons were detected using qPCR assay. (B) Protein levels of TNF-α, IL-1β, and IL-6 in colons were detected using ELISA. (C) Representative Western blots of COX2 and iNOS in colonic tissues. (D) Representative Western blots of p-IκBα, IκBα, p-p65, and p65 in colonic tissues. (E) Cells of colonic tissues were extracted from mice in each group at day 7 and CASP1 activation in CD11b^+^ cells was analyzed by FACS. Data were presented as the mean ± SD and represented 1 of at least 2 independent experiments with consistent results. One-way ANOVA with Tukey's multiple comparisons test (A-E) was used to calculate statistical significance (*p< 0.05, **p < 0.01, ***p < 0.001, and ****p < 0.001).

Proinflammatory cytokines play a critical role in DSS-induced colitis injury as significant elevation of TNF-α, IL-1β, and IL-6, at both mRNA **(Figure 2A)** and protein levels **(Figure 2B)**, was observed by QRT-PCR and ELISA. We investigated whether the protection of DSF+Cu^2+^ in DSS-induced colitis was correlated with reduced proinflammatory cytokine production and found significantly reduced cytokine levels after treatment with DSF and Cu^2+^. Activation of NF-κB plays an essential role in the transcriptional induction of various genes involved in inflammation. As compared to the normal group, DSS-challenged mice exhibited intense canonical NF-κB signaling activation in colonic tissues, indicated by elevated phosphorylation of NF-κB (IκB) α and p65. Remarkably, DSF and Cu^2+^ inhibited canonical NF-κB signaling activation in colonic tissues, as evidenced by decreased phosphorylation of IκBα and p65 **(Figure 2D)**. Also, the expression of NF-κB downstream proteins, inducible NO synthase (iNOS), and cyclooxygenase-2 (COX2) was inhibited by DSF and Cu^2+^ **(Figure 2C)**. In addition, NLRP3-inflammasome-derived CASP1 activation in colonic tissues from DSF and Cu^2+^-treated mice was also reduced **(Figure 2E)**. Collectively, these results suggested that DSF+Cu^2+^ suppressed proinflammatory cytokine production mainly by constraining the activation of the canonical NF-κB pathway and CASP1 in colonic tissues.

### DSF+Cu^2+^ reduced DSS-induced macrophage infiltration and their proinflammatory cytokine secretion

**Figure 3:**
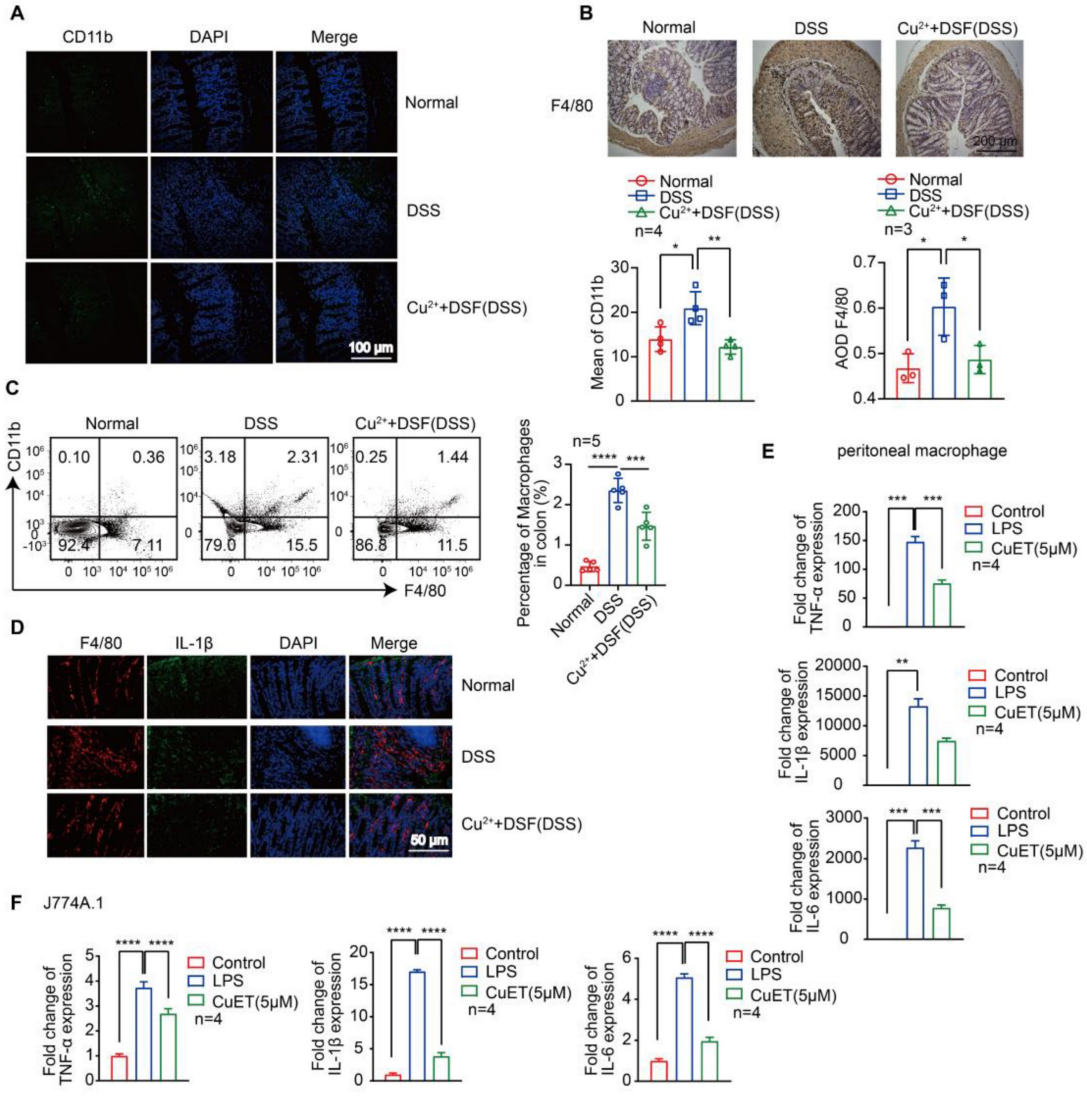
** DSF+Cu^2+^ reduced DSS-induced macrophage infiltration and their proinflammatory cytokine secretion.** (A) Sections of colonic tissue were immune-stained with DAPI (blue) and anti-CD11b-FITC (green) and observed by confocal laser-scanning microscopy (scale bar, 100 μm). (B) Representative immunostaining images of colon sections stained for F4/80 (scale bar, 200 μm). (C) Percentage of F4/80^+^ CD11b^+^ macrophages in colonic tissues. (D) Representative immunofluorescence images of F4/80^+^ IL-1β cells in colonic tissues (scale bar, 50 μm). Green: positive staining of IL-1β; Red: positive staining of F4/80. (E, F) Peritoneal macrophages and J774A.1 cells were pretreated with CuET for 4 h and left or stimulated with LPS for 24 h. Subsequently, mRNA levels of TNF-α, IL-1β, and IL-6 in peritoneal macrophages and J774A.1 cells were detected using qPCR assay. Data were presented as the mean ± SD and represented 1 of at least 2 independent experiments with consistent results. One-way ANOVA with Tukey's multiple comparisons test (A-C, E-F) was used to calculate statistical significance (*p< 0.05, **p < 0.01, ***p < 0.001, and ****p < 0.001).

Most of the pro-inflammatory cytokines, which play a key role in DSS-induced colonic lesions, are produced by macrophages 29-31. We examined macrophage infiltration in colonic tissues to elucidate the mechanism by which DSF and Cu^2+^ regulate intestinal inflammation and recovery. CD11b is expressed in dendritic cells, macrophages, monocytes, and eosinophils. We detected infiltrating CD11b^+^ cells in colonic samples from DSS-induced mice that were predominantly located in the mucosa of the lesion site. In contrast, fewer infiltrating cells were detected in colonic samples from mice treated with DSF and Cu^2+^ **(Figure 3A)**. Subsequently, the accumulation of macrophages in colonic tissues was examined by immunohistochemistry and flow cytometry. The F4/80 expression level was relatively high in DSS-induced colonic tissue and decreased after DSF and Cu^2+^ treatment **(Figure 3B-C)**, and this inhibition was associated with the suppression of inflammatory cytokines in the colon.

We further studied IL-1β expression in colonic macrophages *in vivo*. Immunofluorescence studies showed that DSF+Cu^2+^ could reduce the secretion of IL-1β in colonic macrophages **(Figure 3D)**. DSF is metabolized into diethyldithiocarbamate (DTC) *in vivo* and forms complexes with metal ions, particularly with Cu^2+^ to form DTC-Cu^2+^ complex, CuET 32, 33. We investigated if DSF and Cu^2+^ regulated anti-inflammatory activity by treating mouse peritoneal macrophages and J774A.1 cells with CuET, and then stimulating with LPS. Finally, mRNA of pro-inflammatory factors was detected by QRT-PCR. The results showed that CuET significantly attenuated LPS-induced mRNA levels of TNF-α, IL-1β, and IL-6 **(Figure 3E-F)**, consistent with its anti-inflammatory effect on the DSS-induced colitis model. Thus, DSF+Cu^2+^ treatment could reduce macrophage infiltration and decrease the expression of pro-inflammatory cytokines in macrophages.

### DSF+Cu^2+^ inhibited NF-κB signaling and secretion of IL-1β derived from NLRP3 inflammasome

**Figure 4:**
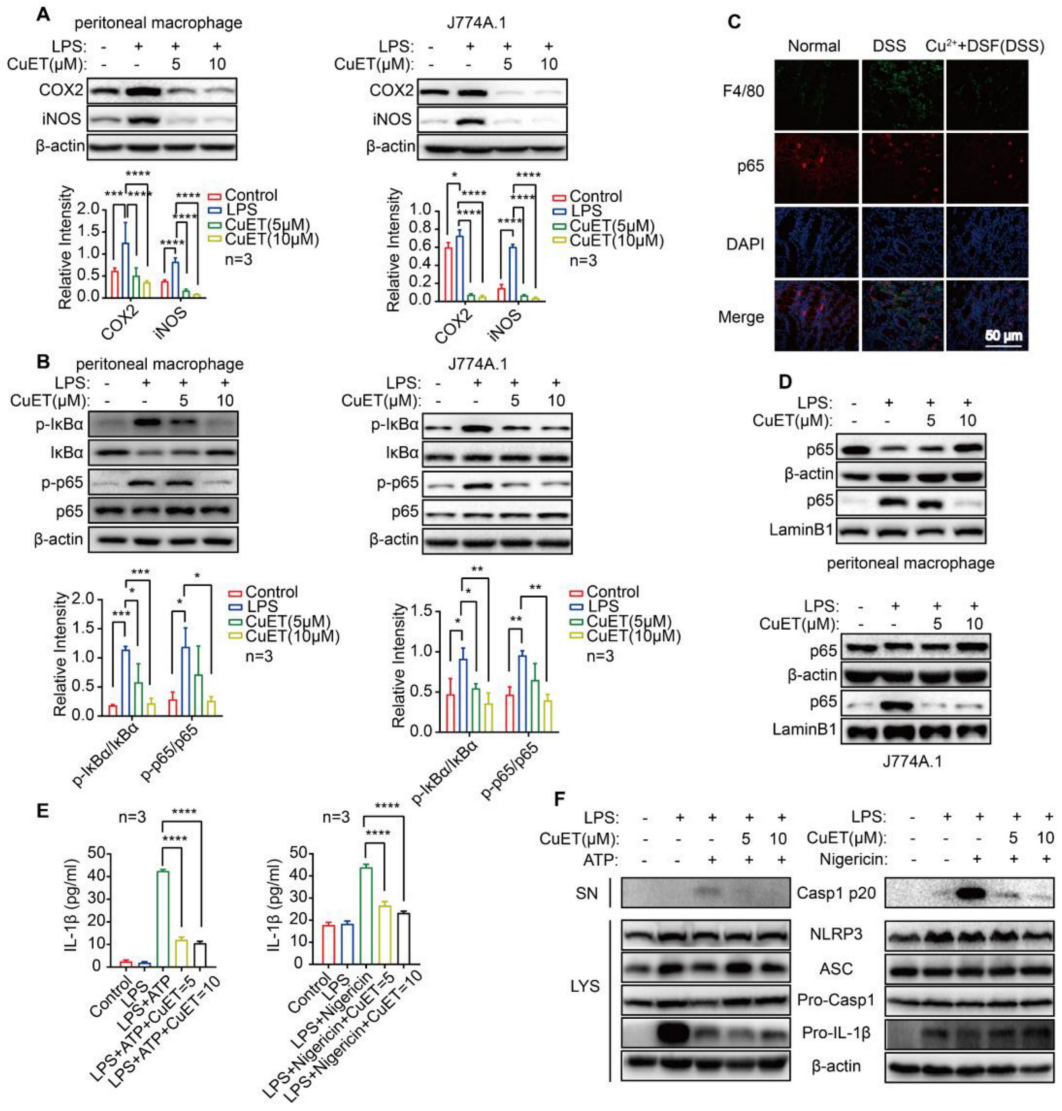
** DSF+Cu^2+^ inhibited NF-κB signaling and secretion of IL-1β derived from NLRP3 inflammasomes.** (A) Peritoneal macrophages and J774A.1 cells were pretreated with CuET for 4 h and left or stimulated with LPS for 24 h, immunoblot and densitometry analyses of iNOS and COX2 in the whole-cell extracts were shown. (B, D) Peritoneal macrophages and J774A.1 cells were pretreated with CuET for 4 h and left or stimulated with LPS for 90 min. (B) Immunoblot and densitometry analyses of the indicated proteins and phosphorylated (p-) proteins in the whole-cell extracts of peritoneal macrophages and J774A.1 cells. (C) Representative immunofluorescence images of F4/80^+^ p65 cells in colonic tissues (scale bar, 50 μm). Green: positive staining of F4/80; Red: positive staining of p65. (D) Immunoblot analyses of the indicated proteins in the cytoplasmic and nuclear extracts of peritoneal macrophages and J774A.1 cells. (E, F) LPS primed-J774A.1 cells were treated with CuET (5 μM and 10 μM) for 4 h and stimulated with nigericin or ATP for another 1 h or 45 min. (E) Cell culture supernatant (SN) was analyzed for IL-1β concentration by ELISA. (F) Immunoblot analyses of culture supernatants (SN) and lysates (LYS) were shown. Data were presented as the mean ± SD and represented 1 of at least 2 independent experiments with consistent results. One-way ANOVA with Tukey's multiple comparisons test (A, B, and E) was used to determine statistical significance (*p< 0.05, **p < 0.01, ***p < 0.001, and ****p < 0.001).

Macrophage activation affects many inflammatory pathways. In an effort to understand the anti-inflammatory effects of DSF+Cu^2+^, we investigated the molecular mechanism of DSF+Cu^2+^ using CuET. First, we found that LPS induced the expression of iNOS and COX2 in peritoneal macrophages and J774A.1 cells, while CuET reduced the expression of inflammatory markers **(Figure 4A)**. We also observed that LPS induced whereas CuET decreased IκB and p65 phosphorylation in J774A.1 cells and peritoneal macrophages **(Figure 4B)**. When macrophages were stimulated with LPS, p65 trans-located from the cytoplasm to the nucleus, however, this translocation was blocked in the CuET group **(Figure 4D)**. Also, the expression of p65 in colonic macrophages was decreased *in vivo* **(Figure 4C)**.

The murine macrophage cell line J774A.1 expressing NLRP3, caspase-1, and ASC has been widely reported as a cellular model to study NLRP3 inflammasome activation 34, 35. We assessed whether CuET could inhibit inflammasome activation by treating J774A.1 with LPS to up-regulate inflammasome components and substrates, including NLRP3 and IL-1β. Then CuET was added to the cell culture prior to stimulation with inflammasome agonists. We chose NLRP3 agonists ATP and nigericin as inflammasome activation could be rapidly assessed after stimulation (30-45 min). ELISA results showed that CuET produced a concentration-dependent inhibition of IL-1β secretion **(Figure 4E)** and a dose-dependent decrease of caspase-1 p20 in supernatants from CuET-treated cells was observed by Western blotting **(Figure 4F)**. Interestingly, CuET did not significantly reduce the expression of inflammasome components **(Figure 4F)**. To further demonstrate that DSF+Cu^2+^ could inhibit NF-κB activity and NLRP3-secreted IL-1β and CASP1 activation, we performed the experiment using THP-1 and BMDMs, and the results were consistent **(Figure S4)**. Hence, DSF+Cu^2+^ could inhibit NF-κB activity and secretion of IL-1β associated with NLRP3 inflammasome and inhibit the activation of CASP1 mediated by NLRP3 inflammasome.

### DSF+Cu^2+^ reduced IL-17 secretion by CD4^+^ T cells

**Figure 5:**
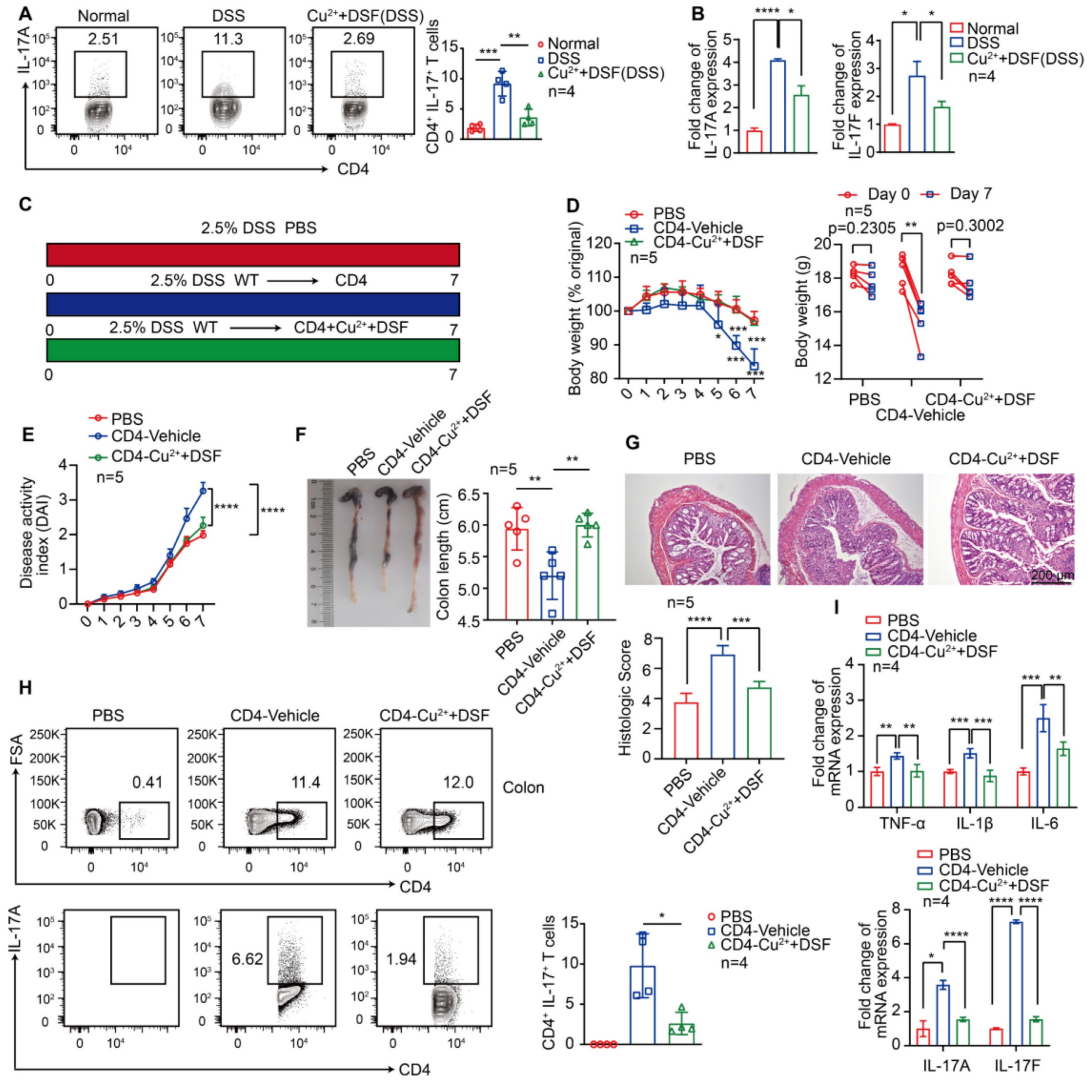
** DSF+Cu^2+^ reduced IL-17 secretion by CD4^+^ T cells.** (A, B) Mice were orally administered with 2.5% DSS and DSF+Cu^2+^ for 7 consecutive days. Mice were then sacrificed, and colons were collected. (A) Percentages of Th17 cells. (B) mRNA levels of IL-17(A) and IL-17(F) were detected using qPCR assay. (C) TCRβ^-/-^ mice were transferred with PBS or naive CD4^+^ T cells pretreated with DSF and Cu^2+^ (CD4-Cu^2+^+DSF) or not (CD4-Vehicle), and were orally administered with 2.5% DSS. (D) Body weight change. (E) DAI score. (F) Colon length. (G) Histological changes were detected using H&E staining (scale bar, 200 μm). (H) Detection of transferred CD4^+^ T cells and percentage of IL-17A^+^ cells in colonic tissues. (I) mRNA levels of TNF-α, IL-1β, IL-6, IL-17(A), and IL-17(F) were detected using qPCR assay. Data were presented as the mean ± SD and represented 1 of at least 2 independent experiments with consistent results. One-way ANOVA with Tukey's multiple comparisons test (A-B and D-I) was used to determine statistical significance (*p< 0.05, **p < 0.01, ***p < 0.001, and ****p < 0.001).

Activation of antigen-presenting cells, such as macrophages, stimulates the immune response mechanism by presenting antigens to T cells. Because immune regulation is crucial for the development and progression of colitis, especially IL-17 secreted by CD4^+^ T cells 36, we investigated the effect of DSF and Cu^2+^ on IL-17 secretion by CD4^+^ T lymphocytes following DSS treatment. The results showed that DSF and Cu^2+^ significantly decreased the proportion of IL-17^+^ CD4^+^ T cells in DSS-induced colitis **(Figure 5A)**. DSS caused a significant increase of IL-17(A) and IL-17(F) mRNA levels, which was largely abolished by DSF and Cu^2+^ treatment **(Figure 5B)**. We assessed the effect of DSF+Cu^2+^ on Th17 cell differentiation by using CuET in the mouse naive CD4^+^ T cells undergoing Th17 differentiation *in vitro* and found that the treatment effectively reduced the percentage of Th17 cells **(Figure S5)**. Since previous studies showed that αβ T cells synergistically enhanced IMQ-induced IL-17A responses in inflammatory diseases, such as psoriasis 37, we next tested whether this response might be involved in DSS-induced colitis. TCRβ^-/-^ mice exhibited remission of pathological features of colitis and the expression of proinflammatory factors in the colon compared with WT mice treated with DSS **(Figure S6)**. The role of DSF and Cu^2+^ in regulating CD4^+^ T cells in the development of DSS-induced colitis was investigated. CD4^+^ T cells were pretreated with DSF and Cu^2+^ (CD4-Cu^2+^+DSF) or the vehicle (CD4-Vehicle) and transferred cells into TCRβ^-/-^ mice treated with DSS **(Figure 5C)**. On day 7, the percentage of CD4^+^ T cells in the spleen of mice transferred with naive CD4^+^ T cells reached 10% **(Figure S7)**. TCRβ^-/-^ mice transferred with CD4-Vehicle T cells developed DSS-induced typical colitis disease, characterized by the pathological features of colitis, including weight loss, increased DAI score, colonic shrinkage, epithelial cell damage, goblet cell loss, crypt swelling and destruction, and inflammatory cell infiltration observed by HE staining **(Figure 5D-G)**. In contrast, TCRβ^-/-^ mice injected with CD4-Cu^2+^+DSF T cells showed significant improvement. Furthermore, the secretion of IL-17 by CD4^+^ T cells in the colon of mice transferred with CD4-Cu^2+^+DSF T cells was inhibited compared with mice transferred with CD4-Vehicle T cells **(Figure 5H)**. TCRβ^-/-^ mice transferred with CD4-Cu^2+^+DSF T cells had lower IL-17A and IL-17F mRNA levels in DSS-induced colitis than those transferred with CD4 control. The same was true for proinflammatory cytokines TNF-α, IL-1β, and IL-6 **(Figure 5I)**. These results showed that DSF and Cu^2+^ might regulate IL-17 production by CD4^+^ T cells.

### DSF+Cu^2+^ improved intestinal microbiota of mice with DSS-induced colitis

**Figure 6:**
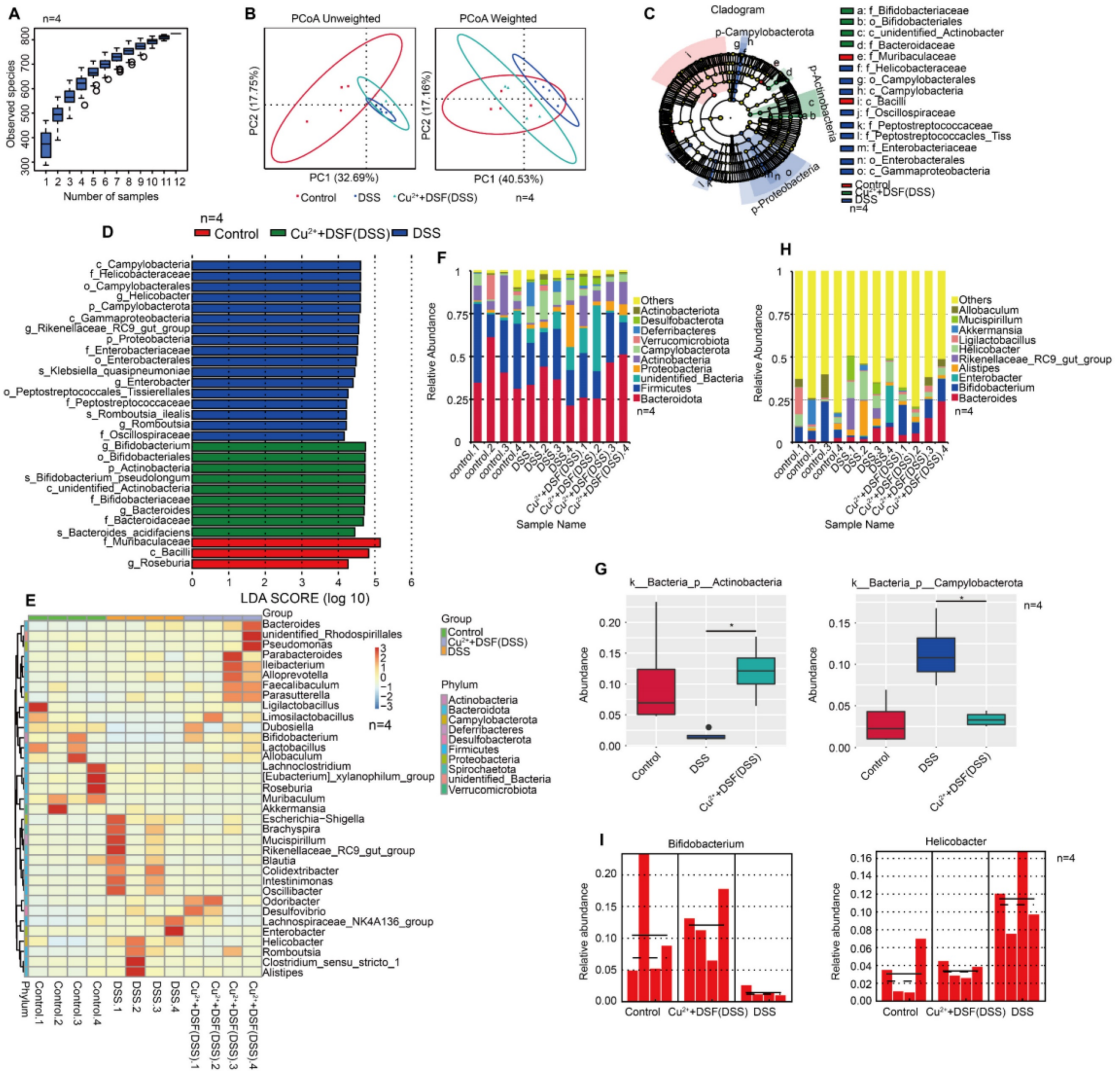
** DSF+Cu^2+^ improved intestinal microbiota of mice with DSS-induced colitis.** (A) Sample number and species richness were estimated from a species accumulation boxplot. (B) PCoA using unweighted-UniFrad and weighted-UniFrad of beta diversity were shown. (C) Taxonomic cladogram from LEfSe, depicting taxonomic association of microbiome communities between Control, DSS, and DSF+Cu^2+^+DSS groups. Each node represented a specific taxonomic type. Yellow nodes denoted taxonomic features that were not significantly different between Control, DSS, and DSF+Cu^2+^+DSS groups. Green nodes denoted taxonomic types with more abundance in DSF+Cu^2+^+DSS group, while the blue nodes represented taxonomic types more abundant in the DSS group and the red nodes represented taxonomic types more abundant in the Normal group. (D) LDA score computed from features differentially abundant between Control, DSS, and DSF+Cu^2+^+DSS groups. The criterion for feature selection was log LDA score > 4. (E) Heatmap of selected most differentially abundant features at the genus level. (F) Bar plots of the phylum taxonomic levels in Control, DSS, and DSF+Cu^2+^+DSS groups. Relative abundance was plotted for each sample. (G) Relative abundance of microbial flora at the phylum level. (H) Bar plots of the genus taxonomic levels in Control, DSS, and DSF+ Cu^2+^+DSS groups. Relative abundance was plotted for each sample. (I) Relative abundance of genus Bifidobacterium and Helicobacter in each sample was displayed by bar plots. Data were presented as the mean ± SD and represented 1 of at least 2 independent experiments with consistent results. One-way ANOVA with Tukey's multiple comparisons test (G) was used to determine statistical significance (*p< 0.05, **p < 0.01, ***p < 0.001, and ****p < 0.001).

In UC, intestinal flora plays a fundamental role in innate and adaptive immunity. Since gut microbiota are involved in colitis development, we investigated if DSF and Cu^2+^ treatment altered the microbiome. We performed high-throughput gene sequencing analysis of 16S rRNA in fecal bacterial DNA isolated from Control, DSS, and DSF+Cu^2+^+DSS mice. Sample size and species richness were estimated from boxplots of species accumulation **(Figure 6A)**. Beta-diversity using unweighted-UniFrac distance and weighted-UniFrac distance algorithms, was performed to generate the principal coordinate analysis (PCoA) for detecting microbiome diversity **(Figure 6B)**. The clear cluster separation among the operational taxonomic units (OTUs) revealed distinct community structures among the three groups, suggesting that these communities were distinct in compositional structure. We then analyzed the gut microbiota to investigate further potential compositional differences between the Control, DSS, and DSF+Cu^2+^+DSS groups. We performed high-dimensional class comparisons using linear discriminant analysis (LDA) of effect size (LEfSe) to confirm which bacteria were altered by DSF and Cu^2+^ treatment and, in turn, affected disease progression in DSS-induced colitis. Significant differences in the dominance of bacterial communities among the three groups were detected **(Figure 6C-D)**. Our results showed that Campylobacterota (the phylum, the class and the order Campylobacterales) and Helicobacteraceae (the family and the genus Helicobacter) were the key types of bacteria contributing to gut microbiota dysbiosis in the DSS group. In the DSF+Cu^2+^+DSS groups, Bifidobacteriales (the order, the family and the genus Bifidobacterium), Bacteroidaceae (the family and the genus Bacteroides) and Actinobacteria exhibited a relative enrichment, which might be associated with the DSF+Cu^2+^ -mediated alleviation of colitis.

Based on genus-level OTU abundances, we analyzed gut microbiota with a comparative heatmap between the three groups **(Figure 6E)**. Actinobacteria abundance decreased in the phylum after DSS treatment, but increased significantly in the DSF and Cu^2+^ groups. On the contrary, Campylobacterota abundance increased after DSS treatment, but significantly reduced in the DSF and Cu^2+^ group **(Figure 6F-G)**. The taxonomic composition was also analyzed at class/order/family/ species level **(Figure S8)**. Among the genera, Bifidobacterium abundance decreased after DSS treatment but increased significantly with the treatment of DSF and Cu^2+^, and Helicobacter increased after DSS treatment, but decreased significantly with DSF and Cu^2+^ treatment **(Figure 6H-I)**. Many previous studies have shown that Actinobacteria are inversely correlated with colon injury 38 and Bifidobacterium longum is a species of commensal bacteria present in the human gastrointestinal tract. Both animal and clinical trials have reported that Bifidobacterium longum could reduce chronic mucosal inflammation in UC patients and prevent the occurrence of experimental colitis induced by DSS 39, 40. Campylobacter, a zoonotic pathogen, is the leading cause of human bacterial enteritis 41 and Helicobacter has been recognized as a major risk factor for gastritis, peptic ulcers, gastric cancer, and gastric mucous-associated lymphoma 42, 43. Our data suggested that DSS induction increased harmful bacteria in the gut, but DSF+Cu^2+^ treatment increased beneficial gut bacteria.

## Discussion

UC is a debilitating inflammatory disease of the intestine. Its clinical treatment is mainly based on anti-inflammatory drugs (including steroid hormones, salicylic acid drugs, and glucocorticoids) and immunosuppressive agents combined with surgery 44. Data from several studies show that the clinical response rate of anti-inflammatory drugs and immunosuppressants is not high, and their serious side effects and sequelae also bring more disease burden to patients. Therefore, there is an urgent need to develop new treatment options.

DSF is an anti-alcohol drug that produces serious discomfort, and side effects. DSF is inexpensive, and its pharmacokinetics, safety, and tolerability are well established at the doses recommended by FDA 8. In this study, we investigated the anti-inflammatory effect of DSF+Cu^2+^ on colitis. It has been shown that mice pretreated with copper gluconate for 6 hours and then treated with DSF showed improved survival of mice LPS-induced sepsis compared to mice treated with DSF alone. This suggested that DSF given after LPS partially protected mice and administration of Cu^2+^ may have improved its activity 14. Therefore, we used this method of administration to investigate the relief of DSS-induced colitis by DSF+Cu^2+^. Our results showed that DSF+Cu^2+^ had a protective effect on DSS-induced colitis, manifested by significantly improved body weight, colonic length, histological score, reduced DAI score, and increased expression of MUC2, ZO-1 and occludin. Previous studies have shown that disulfiram alone is not effective in treating DSS-induced colitis, but when combined with lactoferrin, it can significantly alleviate the condition 45. In this study, we investigated whether Cu^2+^ alone has the protective effects or enhance the effectiveness of DSF in treating colitis. Our findings revealed that compared to Cu^2+^ or DSF alone, the combination of DSF+Cu^2+^ significantly relieved DSS-induced colitis. On the other hand, it also indicated that our drug administration method was effective. These results suggested that DSF+Cu^2+^ can relieve symptoms of colitis and protect the intestinal barrier.

Macrophages play an important role in regulating the homeostasis of the intestinal immune microenvironment. In past studies, macrophages have been shown to be extensively involved in the initiation and development of IBD-related inflammation 46-49. The contribution of pro-inflammatory cytokines (TNF-α, IL-6, IL-1β, etc), iNOS, and COX2 in UC and DSS-induced models has been established 50. NF-κB signaling plays an important role in promoting inflammation responses in macrophages and can be activated by various stimuli, such as LPS. Activation of the NF-κB pathway could lead to the transcription of proinflammatory genes, resulting in increased proinflammatory factors 51. UC could be improved by inhibiting NF-κB activity via phosphorylation of the inhibitor of NF-κB (IκBα or IκBβ) or using protease inhibitors and antisense oligonucleotides of p65 *in vivo* 52. In our study, DSF+Cu^2+^ inhibited the activity of NF-κB in DSS-induced colitis and LPS-induced peritoneal macrophages, J774A.1, THP-1, and BMDMs, resulting in down-regulation of proinflammatory factors. Thus, DSF+Cu^2+^ inhibited the NF-κB pathway.

Multiple studies have shown that NLRP3 inflammasomes are mainly produced by macrophages and are closely associated with UC 22, 53, 54. Upon activation, NLRP3 proteins polymerize and bind to the ASC adaptor, promoting the recruitment of pro-caspase-1 55, which then clusters and auto-cleaves into its activated form, caspase-1 p10/p20 tetramer. The activated form then triggers caspase-1-dependent processing of pro-IL-1β and allows the secretion of the mature forms of these cytokines, and activated caspase-1 is secreted along with mature IL-1β 56. LPS/ATP-induced or LPS/nigericin-induced macrophages upregulate inflammasome components and substrates, including NLRP3 and IL-1β. Activation of NLRP3 inflammasomes and the production of IL-1β in macrophages have been reported to play a crucial role in the induction of acute colitis 57. Therefore, detecting activated caspase-1 and IL-1β in the supernatant and changes in inflammasome components and substrates were necessary. Our results showed decreased expression of cleaved-caspase-1, cleaved-IL-1β, and secretion of IL-1β in the cell supernatant after DSF+Cu^2+^ treatment, but no consistent effect on the expression of pro-caspase-1, NLRP3, or ASC in cell lysates, demonstrating that DSF+Cu^2+^ inhibited caspase-1 activation and IL-1β secretion.

With increased inflammatory stimulation, the number of antigen-presenting cells in UC increases, including dendritic cells and macrophages 16. Activation of antigen-presenting cells releases cytokines, stimulating T cell homeostasis and causing inflammation in UC patients. Excessive Th17 cell development and IL-17 production are associated with the pathogenesis of several diseases, including autoimmune arthritis, multiple sclerosis, and UC 58, 59. In our study, DSF+Cu^2+^ could reduce IL-17 secreted by Th17 cells in the intestine in DSS-induced colitis. *In vitro* addition of cytokines (TGFβ, IL-1β, IL-6, and IL-23) induces the polarization of naive CD4^+^ T cells, and the expression of IL-17 is reduced after administration. We used TCRβ^-/-^ mice transferred with CD4 or CD4-Cu^2+^+DSF to demonstrate that DSF+Cu^2+^ could regulate the secretion of IL-17 by Th17 cells and plays an important role.

The pathogenesis of UC is related to many inflammatory pathways, including epithelial barrier, symbiotic bacteria, antigen recognition, and immune dysregulation 16. Unlike common inflammatory diseases, dysregulation of gut microbiota plays an indispensable pathogenic role in UC. Intestinal microbes can alter the homeostasis of intestinal immune cells and aggravate colitis in mice 60. Therefore, it is necessary to investigate whether gut microbiotas are involved in the protective effect of DSF+Cu^2+^. The loss of beneficial symbiotic flora and the expansion of pathogenic bacteria were shown in UC patients 61. We used 16S rRNA sequencing to investigate potential changes in microbial diversity and composition following DSF+Cu^2+^ treatment. The results of Alpha diversity measurement based on OTUs showed that the microbiota diversity in DSF+Cu^2+^+DSS group was not significantly better than that in the DSS group. In terms of beta diversity, mice in the DSF+Cu^2+^+DSS group were separated from mice in the DSS group by PCoA, indicating that DSF+Cu^2+^ treatment significantly changed the structure of the biological community. We performed LEfSe analysis between the DSF+Cu^2+^+DSS and the DSS groups to identify potential biomarkers and dominant microbiota mediated by DSF+Cu^2+^ treatment. Importantly, three signature bacterial groups, including Bifidobacterium, Bacteroides and Actinobacteria, showed enrichment in DSF+Cu^2+^+DSS group. Therefore, our results suggested that DSF+Cu^2+^ might modulate gut microbiota dysregulation induced by DSS by promoting the relative abundance of beneficial bacteria. It is possible that DSF+Cu^2+^ affects intestinal immune homeostasis through intestinal flora. However, the exact mechanism needs further research.

In conclusion, DSF+Cu^2+^ could significantly diminish the progression of DSS-induced UC. DSF+Cu^2+^ inhibited the decrease in tight junction proteins, ZO-1, occludin, and MUC2 to protect the intestinal barrier, macrophage activation in colonic tissue, and IL-17 secreted by CD4^+^ T cells, the treatment also reduced the abundance of harmful bacteria while increasing the beneficial bacteria, thereby improving the overall gut microbiota. *In vitro* experiments showed that CuET inhibited the NF-κB pathway and NLRP3-related CASP1 activation and IL-1β secretion. In summary, our results have provided evidence for the rational use of DSF+Cu^2+^ for colonic inflammation, suggesting the potential therapeutic efficacy of the treatment for UC and therefore warrant further exploration.

## Supplementary Material

Supplementary figures and table.

## Abbreviations

ANOVA: one-way analysis of variance
ASC: apoptosis-associated speck-like protein containing a caspase recruitment domain
ATP: adenosine triphosphate
BMDMs: bone marrow-derived macrophages
CASP1: caspase1
CARD: caspase recruitment domain
CD: crohn's disease
COX-2: cyclooxygenase-2
CuET: bis (diethyldithiocarbamate)-copper
DAI: disease activity index
DTC: diethyldithiocarbamate
DSF: disulfiram
DSS: dextran sulfate sodium
ELISA: enzyme-linked immunosorbent assay
FBS: fetal bovine serum
FDA: food and drug administration
H&E: hematoxylin and eosin
IBD: inflammatory bowel disease
IL-6: interleukin-6
IL-17: interleukin 17
IL-1β: interleukin 1 beta
iNOS: inducible NO synthase
LDA: linear discriminant analysis
LPS: lipopolysaccharide
M-CSF: macrophage colony-stimulating factor
MUC2: mucoprotein-2
NF-κB: nuclear factor-kappa B
NLRP3: nucleotide- binding oligomerization domain, leucine-rich repeat and pyrin domain-containing 3
OTUs: operational taxonomic units
PCoA: principal coordinate analysis
PMA: phorbol 12-myristate 13-acetate
PMSF: phenylmethyl sulfonyl fluoride
qPCR: quantitative real-time PCR
TH17: T helper type 17
TNF-α: tumor necrosis factor α
Treg: regulatory T
UC: ulcerative colitis
ZO-1: zonula occluden-1
