# Supplementary material for: Disulfiram with Cu2+ alleviates dextran sulfate sodium-induced ulcerative colitis in mice
Source: Theranostics. 2023 May 8;13(9):2879–95. doi: 10.7150/thno.81571 (PMC10240830; doi:10.7150/thno.81571)
Supplement: Supplementary file 1 — Supplementary figures and table. [file thnov13p2879s1.pdf]

Table S1. Quantitative PCR primer sequences.

Primer name	**Forward Primer** (5' to 3')	**Reverse Primer** (5' to 3')
Mouse TNF-α	CTGAACTTCGGGGTGATCGG	GGCTTGTCACTCGAATTTTGAGA
Mouse IL-1β	GAAATGCCACCTTTTGACAGTG	TGGATGCTCTCATCAGGACAG
Mouse IL-6	CTGCAAGAGACTTCCATCCAG	AGTGGTATAGACAGGTCTGTTGG
Mouse IL-17A	ATGCTGTTGCTGCTGCTGAG	GGAAGTCCTTGGCCTCAGTG
Mouse IL-17F	GGAGGTAGCAGCTCGGAAGA	GGAGCGGTTCTGGAATTCAC
Mouse GAPDH	AGGTCGGTGTGAACGGATTTG	TGTAGACCATGTAGTTGAGGTCA
Human TNF-α	GGACACCATGAGCACTGAAAGC	TGCCACGATCAGGAAGGAGAAG
Human IL-1β	CCACAGACCTTCCAGGAGAATG	GTGCAGTTCAGTGATCGTACAGG
Human IL-6	AATTCGGTACATCCTCGACGGC	GCCAGTGCCTCTTTGCTGCTTT
Human GAPDH	ATGGGGAAGGTGAAGGTCG	GGGGTCATTGATGGCAACAATA

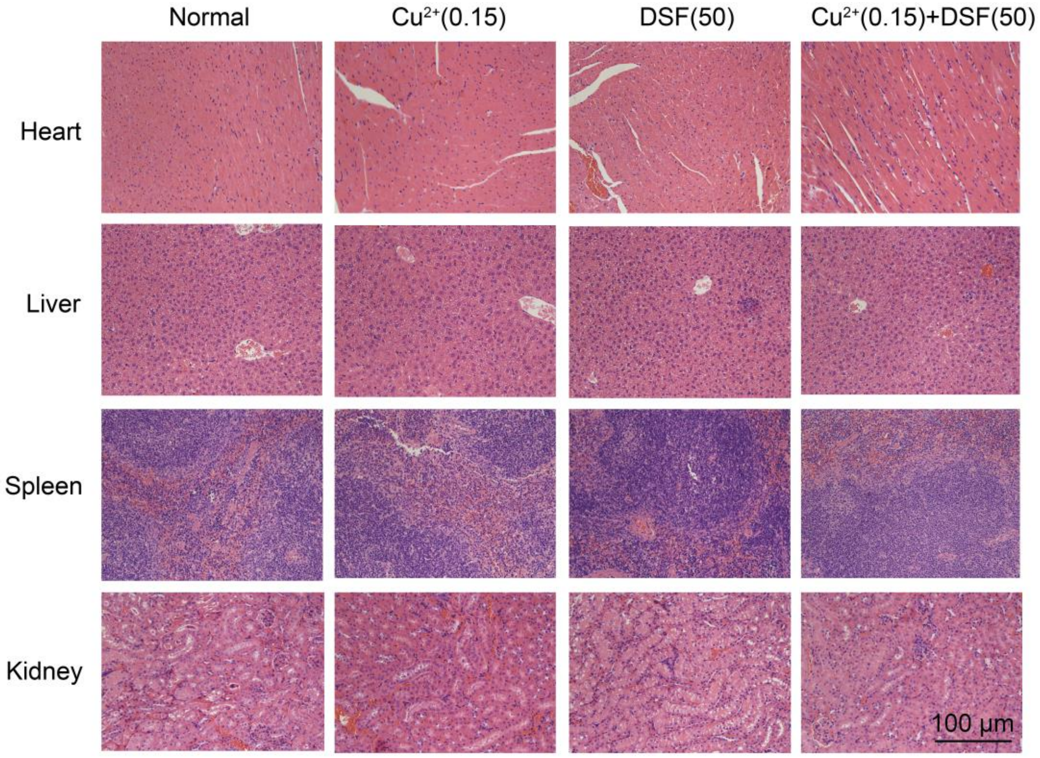

Figure S1. The preliminary evaluation of biosafety. Histological examination of major organs was detected using H&E staining (scale bar, 100 μm) (n = 4).

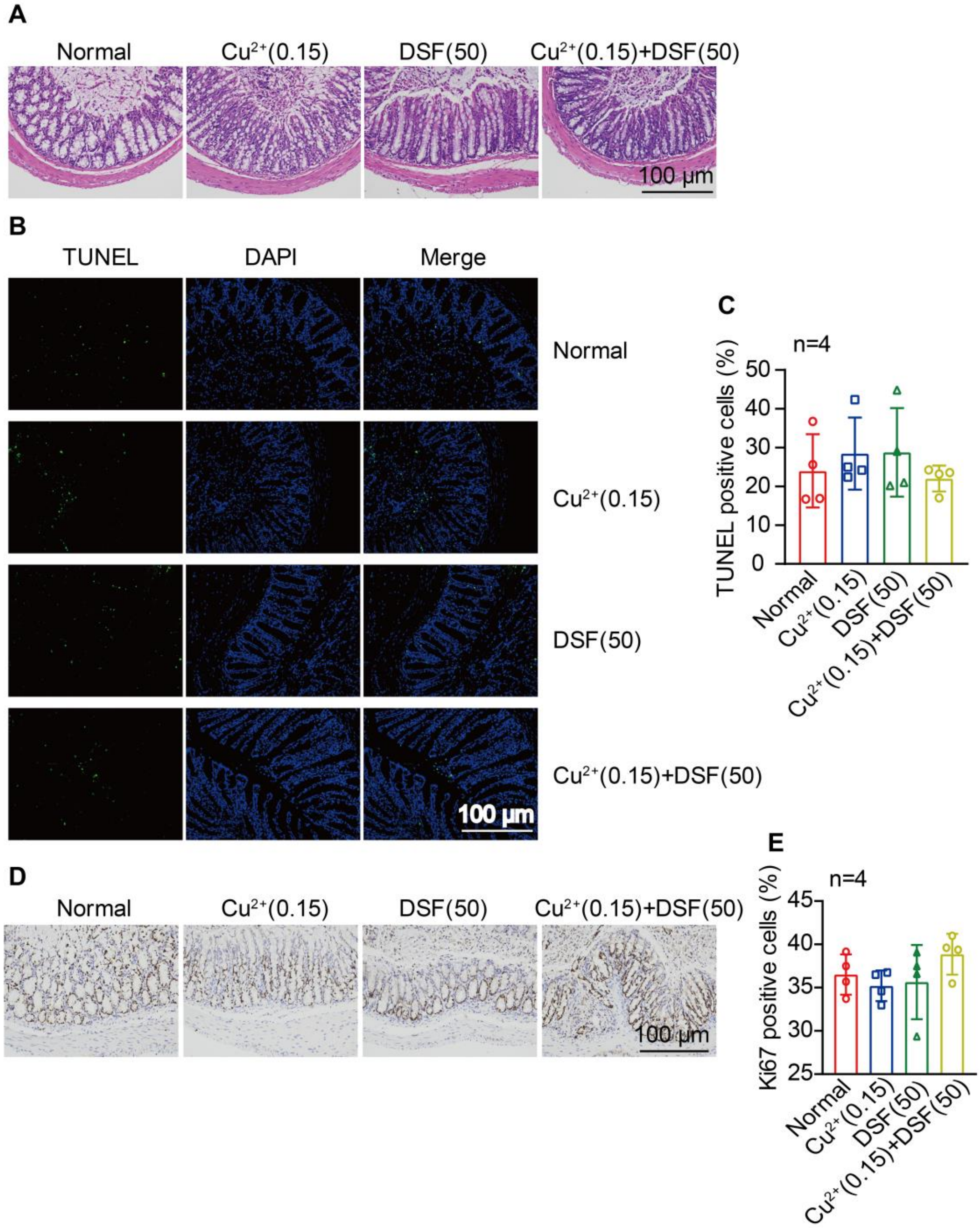

Figure S2. Effect of disulfiram with Cu^{2+} on cell apoptosis and proliferation in colonic tissues. (A) Histological changes were detected using H&E staining (scale bar, 100 µm) (n=4). (B) Representative images for (TUNEL) staining (scale bar, 100 µm). (C) Assessment of the apoptotic cells in each group. (D) Representative immunostaining images for Ki67 (scale bar, 100 µm). (E) Ki67-positive cells analysis.

Data were presented as the mean ± SD and represented 1 of at least 2 independent experiments with consistent results. One way ANOVA with Tukey's multiple comparisons test (C, E) was used to calculate statistical significance (**p*< 0.05, ***p* < 0.01, ****p* < 0.001, and *****p* < 0.001).

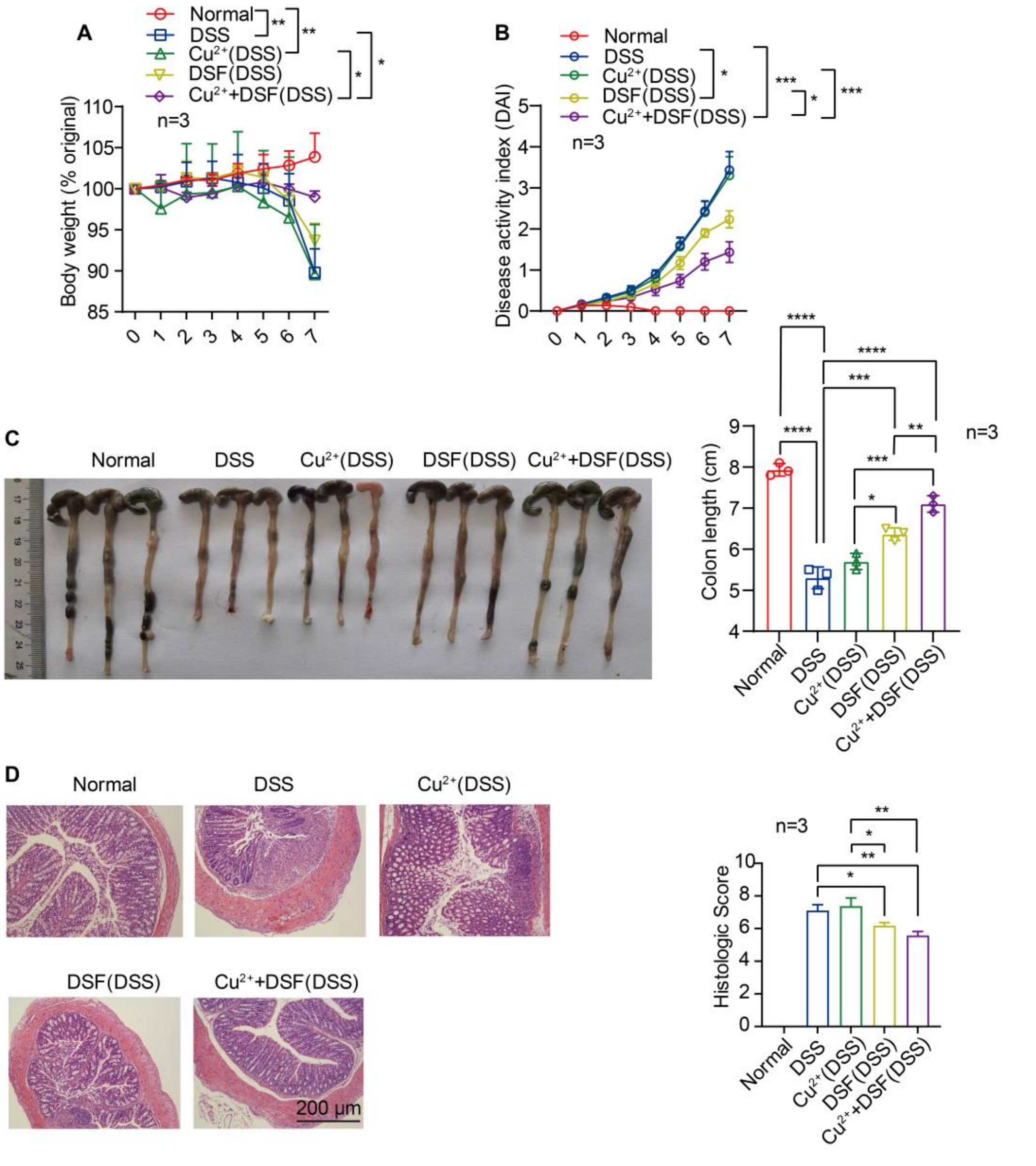

Figure S3. DSF+Cu^{2+} alleviated DSS-induced colitis in mice. (A) Mice were orally administered 2.5% DSS, Cu^{2+}, DSF, or DSF+Cu^{2+} for 7 consecutive days. Mice were then sacrificed, and colons were collected. (A) Body weight change. (B) DAI score.

(C) Colon length. (D) Histological changes were detected using H&E staining (scale bar, 200 μm). Data were presented as the mean ± SD and represented 1 of at least 2 independent experiments with consistent results. One way ANOVA with Tukey's multiple comparisons test (A-D) was used to calculate statistical significance (*p< 0.05, **$p < 0.01$, ***$p < 0.001$, and ****$p < 0.001$).

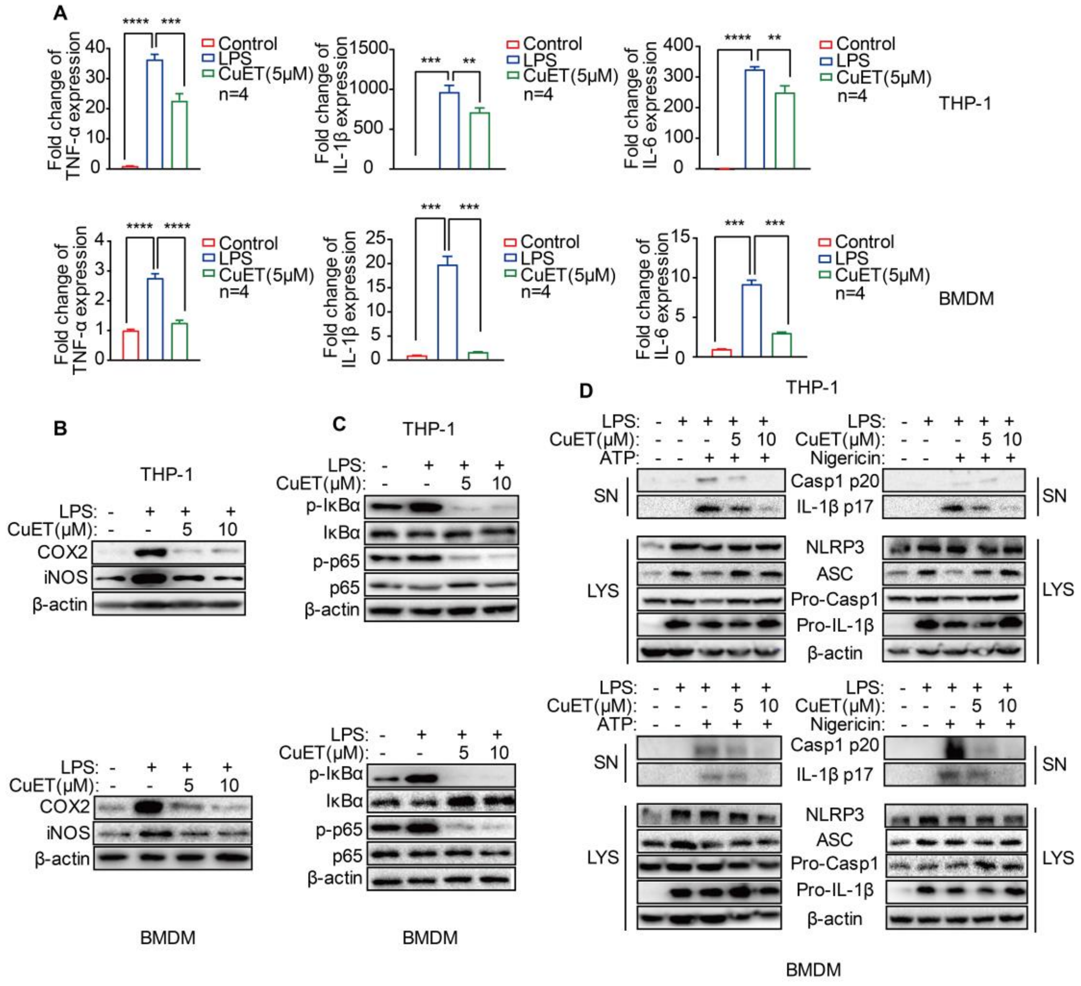

Figure S4. DSF+Cu^{2+} inhibited NF-κB signaling and secretion of IL-1β derived from NLRP3 inflammasomes. (A) THP-1 and BMDMs were pretreated with CuET for 4 h and left or stimulated with LPS for 24 h, mRNA levels of TNF-α, IL-1β, and IL-6 in THP-1 and BMDMs were detected using qPCR assay. (B) THP-1 and BMDMs were pretreated with CuET for 4 h and left or stimulated with LPS for 24 h.

Immunoblot analyses of iNOS and COX2 in the whole-cell extracts were shown. (C) THP-1 and BMDMs were pretreated with CuET for 4 h and left or stimulated with LPS for 90 min. Immunoblot analyses of the indicated proteins and phosphorylated (p-) proteins in the whole-cell extracts of THP-1 and BMDMs. (D) LPS primed-THP-1 and LPS primed-BMDMs were treated with CuET (5 μM and 10 μM) for 4 h and stimulated with nigericin or ATP for another 1 h or 45 min. Immunoblot analyses of culture supernatants (SN) and lysates (LYS) were shown. Data were presented as the mean ± SD and represented 1 of at least 2 independent experiments with consistent results. One way ANOVA with Tukey's multiple comparisons test (A) was used to determine statistical significance (**p*< 0.05, ***p* < 0.01, ****p* < 0.001, and *****p* < 0.001).

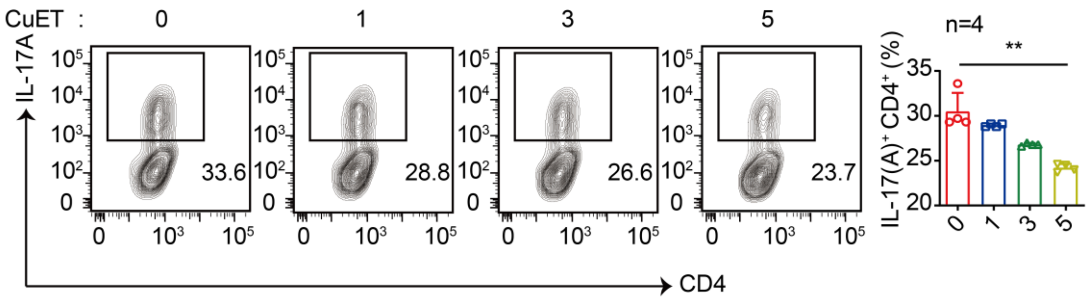

Figure S5. Mouse naive CD4$^+$ T cells were polarized under indicated conditions in vitro for 4 days. Cells were either left untreated or treated with CuET under 1 μM, 3 μM, or 5 μM. Flow cytometry was performed for the percentages of Th17 cells. Data were presented as the mean ± SD and represented 1 of at least 2 independent experiments with consistent results. One way ANOVA with Tukey's multiple comparisons test was used to determine statistical significance (*p< 0.05, **p < 0.01, ***p < 0.001, and ****p < 0.001).

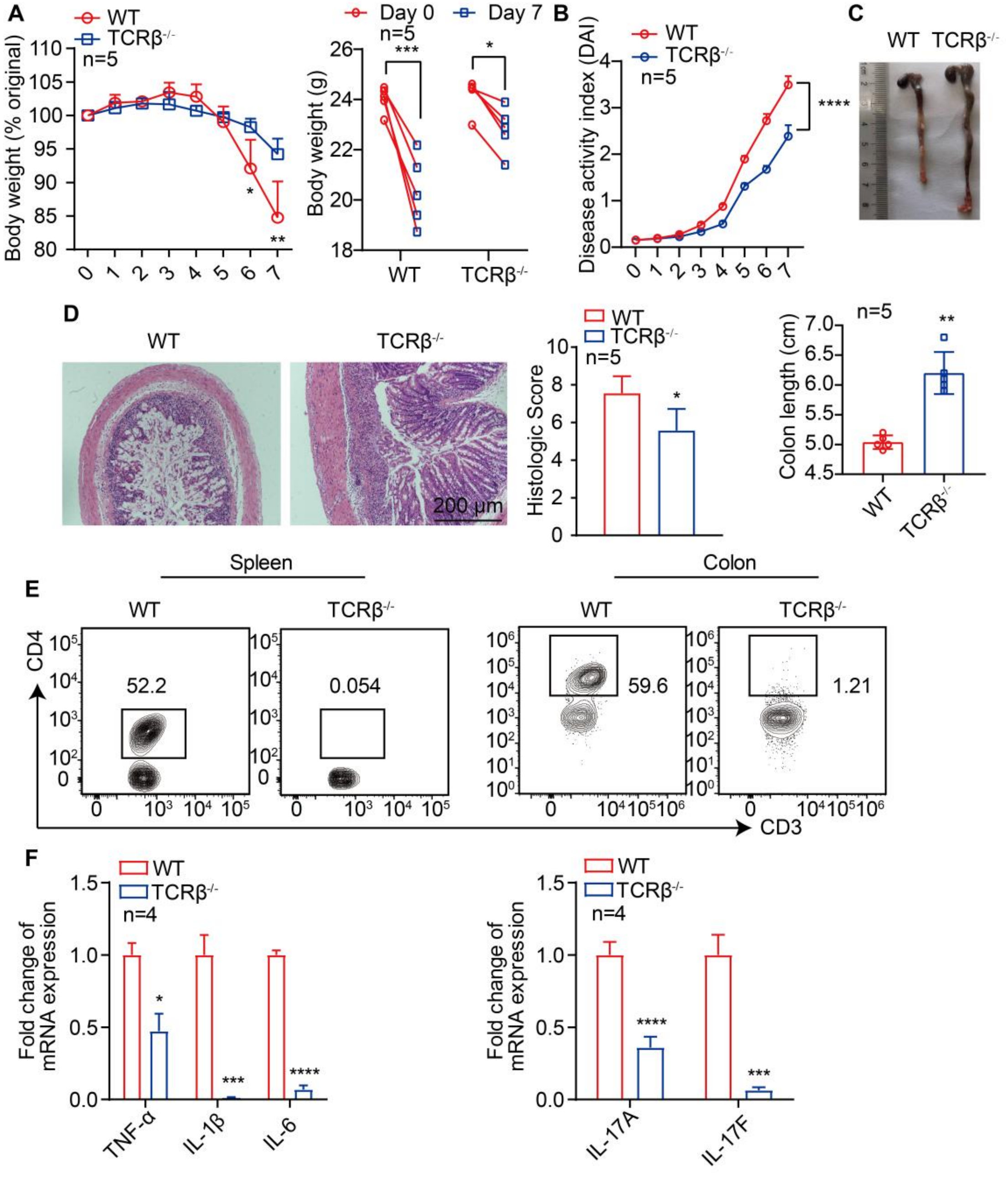

Figure S6. αβ T cells were critical in the pathological phenotype of DSS-induced colitis mice. WT and TCRβ$^{-/-}$ mice were treated with DSS to induce colitis. (A) Body weight change. (B) DAI score. (C) Colon length. (D) Histological changes were detected using H&E staining (scale bar, 200 μm). (E) Percentage of CD4^{+} T cells in the spleen and colonic tissues. (F) Relative mRNA expression of TNF-α, IL-1β, IL-6, IL-17A, and IL-17F in colonic tissues. Data were presented as the mean ± SD and

represented 1 of at least 2 independent experiments with consistent results. 2-tailed, unpaired Student's t test (A-D and F) was used to determine statistical significance (*p< 0.05, **p < 0.01, ***p < 0.001, and ****p < 0.001).

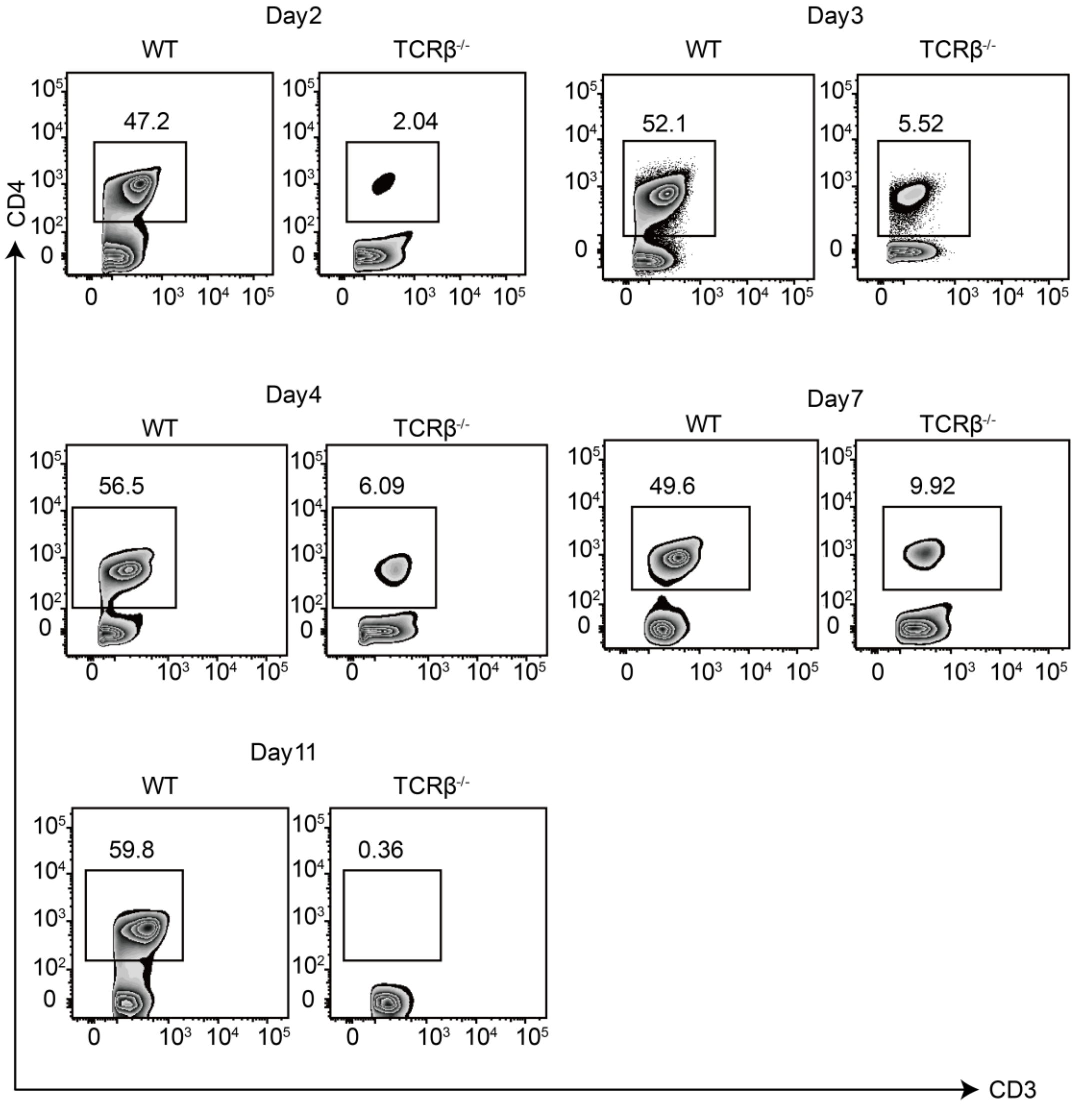

Figure S7. Detection of transferred CD4⁺ T cells in the spleen. TCRβ$^{-/-}$ mice were transferred naive CD4^{+} T cells, which were detected by flow cytometry on Days 2, 3, 4, 7, 11.

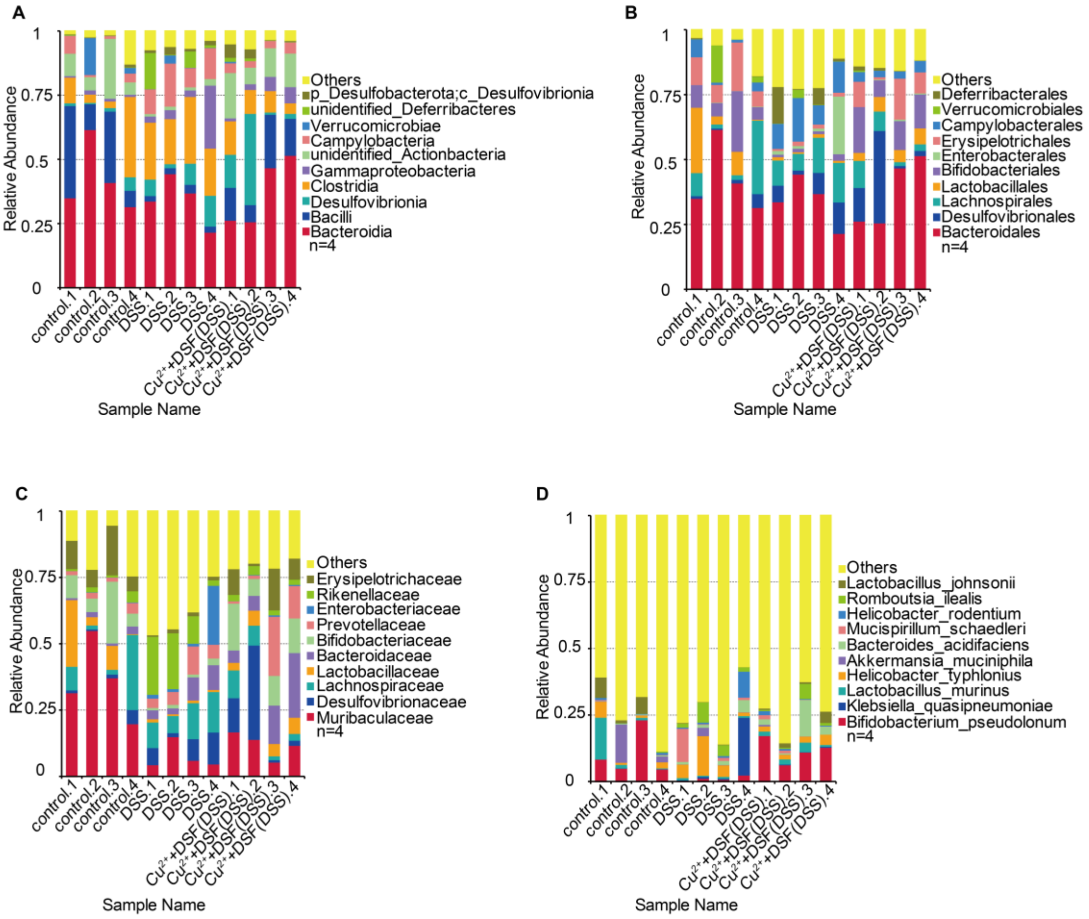

Figure S8. DSF+Cu^{2+} improved the intestinal microbiota of mice with DSS-induced colitis. (A-D) Bar plots of the class (A), order (B), family (C), and species (D) taxonomic levels in Control, DSS, and DSF+Cu^{2+}+DSS groups. Relative abundance was plotted for each sample.

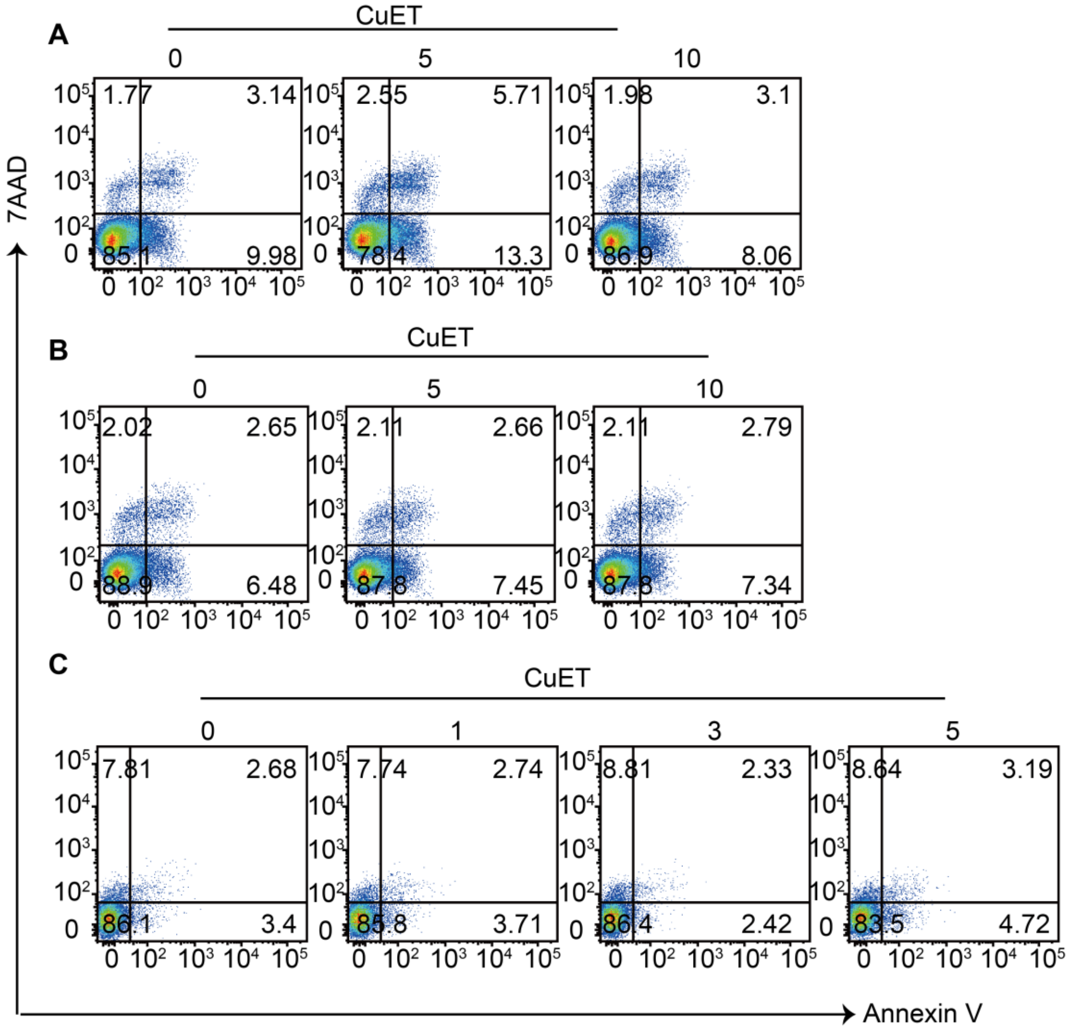

Figure S9. Viability measures of cells treated with CuET in vitro. (A) Peritoneal macrophages were treated with CuET at 0 µM, 5 µM, or 10 µM (n=3). (B) J774A.1 cells were treated with CuET at 0 µM, 5 µM, or 10 µM (n=3). (C) Mouse naive CD4^{+} T cells were polarized under indicated conditions in vitro for 4 days, and then the cells were treated with CuET at 0 µM, 1 µM, 3 µM, or 5 µM (n=4). Cell viability was measured by FACS using 7AAD/Annexin V kit.
